# *PbrMYB21*, a novel MYB protein of *Pyrus betulaefolia*, functions in drought tolerance and modulates polyamine levels by regulating arginine decarboxylase gene

**DOI:** 10.1111/pbi.12708

**Published:** 2017-04-01

**Authors:** Kongqing Li, Caihua Xing, Zhenghong Yao, Xiaosan Huang

**Affiliations:** ^1^ Department of Rural Development Nanjing Agricultural University Nanjing China; ^2^ College of Horticulture State Key Laboratory of Crop Genetics and Germplasm Enhancement Nanjing Agricultural University Nanjing China

**Keywords:** abiotic stress, polyamine biosynthesis, MYB, *Pyrus betulaefolia*, transcriptional regulation

## Abstract

MYB comprises a large family of transcription factors that play significant roles in plant development and stress response in plants. However, knowledge concerning the functions of MYBs and the target genes remains poorly understood. Here, we report the identification and functional characterization of a novel stress‐responsive MYB gene from *Pyrus betulaefolia*. The MYB gene, designated as *PbrMYB21*, belongs to the R2R3‐type and shares high degree of sequence similarity to *MdMYB21*. The transcript levels of *PbrMYB21* were up‐regulated under various abiotic stresses, particularly dehydration. PbrMYB21 was localized in the nucleus with transactivation activity. Overexpression of *PbrMYB21* in tobacco conferred enhanced tolerance to dehydration and drought stresses, whereas down‐regulation of *PbrMYB21* in *Pyrus betulaefolia* by virus‐induced gene silencing (VIGS) resulted in elevated drought sensitivity. Transgenic tobacco exhibited higher expression levels of *ADC* (arginine decarboxylase) and accumulated larger amount of polyamine in comparison with wild type (WT). VIGS of *PbrMYB21* in *Pyrus betulaefolia* down‐regulated ADC abundance and decreased polyamine level, accompanied by compromised drought tolerance. The promoter region of *PbrADC* contains one MYB‐recognizing *cis‐*element, which was shown to be interacted with PbrMYB21, indicating the *ADC* may be a target gene of *PbrMYB21*. Take together, these results demonstrated that *PbrMYB21* plays a positive role in drought tolerance, which may be, at least in part, due to the modulation of polyamine synthesis by regulating the *ADC* expression.

## Introduction

Drought is one of the major abiotic stresses affecting plant growth, development, productivity and geographic distribution (Farooq *et al*., 2009). To cope with this stress, plants have evolved sophisticated mechanisms to adapt to drought stress, ranging from perception of stress signals to modifications of physiological and biochemical responses (Ingram and Bartels, 1996; Pastori and Foyer, 2002). In these regulating processes, plants undergo tremendous molecular changes by reprogramming an array of stress‐responsive genes (Hirayama and Shinozaki, 2010; Liu *et al*., 2014; Osakabe *et al*., 2014; Seki *et al*., 2001). These genes can be generally classified into two main groups based on their products, effector molecules and regulator molecules. Among various regulatory genes, transcription factors (TFs) act as significant coordinators to transduce stress signals and to orchestrate expression of functional genes that play direct role in preventing plants from stress‐associated damages (Ren *et al*., 2010). Therefore, genetic engineering of stress‐associated TFs has been proposed to be a robust strategy for improving the stress tolerance of crop plants (Sreenivasulu *et al*., 2007; Thomashow *et al*., 2001; Yu *et al*., 2012; Zhao *et al*., 2016).

Plants modulate a battery of functional genes involved in the synthesis of various metabolites that resist the abiotic stresses. The most common polyamines (PAs), primarily diamine putrescine (Put), triamine spermidine (Spd) and tetramine spermine (Spm), are low molecular weight aliphatic polycations that are ubiquitously present in living organisms, which are a group of aliphatic polycations that are ubiquitously distributed in higher plant. Accumulation of PAs serves as a metabolic hallmark under drought stresses in a large number of plants (Groppa and Benavides, 2008; Yang *et al*., 2007). It has also been suggested that polyamines act as osmotic regulators and scavengers of ROS (Drolet *et al*., 1986; Evans and Malmberg, 1989). Thus, it is conceivable that higher accumulation of polyamines through stimulated *de novo* biosynthesis can be conducive to ameliorating stress‐induced damage. This implies that ADC pathway plays a key role in stress tolerance, as revealed by following experimental data. For example, previous studies demonstrated that overexpression of the *ADC2* gene led to enhance drought tolerance in *Arabidopsis thaliana* (Alcazar *et al*., 2010). In an earlier work, elevation of *ADC* transcript and endogenous polyamine levels by overexpression of polyamine biosynthetic genes has been demonstrated to enhance abiotic stresses tolerance in various plants (Huang *et al*., 2015a,b; Shi *et al*., 2010; Wang *et al*., 2011). In another study, PtADC was reported to be a potential target of PtrABF, which can specifically recognize the abscisic acid (ABA) response element within the promoter of PtADC (Zhang *et al*., 2015). WRKY70 was demonstrated to interact with W‐box elements within the promoter of a Fortunella crassifolia ADC gene (Gong *et al*., 2015). Recently, a stress‐responsive trifoliate orange NAC family TF, PtrNAC72, was reported to be a repressor of putrescine biosynthesis and may negatively regulate the drought stress response, via the modulation of putrescine‐associated reactive oxygen species homoeostasis (Wu *et al*., 2016). All of these results suggest that the *ADC* gene is a promising candidate for enhancing stress tolerance and may be an important gene for molecular breeding of stress‐tolerant plants.

The MYB transcription factors comprise a large gene family in plants. Proteins of this family contain a highly conserved MYB domain at the N‐terminus, comprising one to four imperfect repeats (Lipsick, 1996). Each repeat contains 50–53 amino acid residues, which form each domain into three α‐helices. Analysis of the three‐dimensional structure of the MYB domain has shown that the second and third helices of each repeat have three regularly spaced tryptophan (W) residues forming a helix–turn–helix (HTH) structure with a hydrophobic core, while the third helix directly interacts with the major groove of the target DNA. Based on the number of imperfect adjacent repeats (R1, R2, R3) in the MYB domain, the MYB proteins are primarily classified into four major subgroups, R1R2R3‐type MYB (MYB3R, three repeats), R2R3‐type MYB (two repeats), MYB1R and 4R‐like MYB protein (4R‐MYB). MYB proteins appear to be widespread in plants, but no homologue has been identified thus far in other eukaryotes. The majority of plant MYB genes belong to the R2R3 types, although more than 100 members of this family have been suggested in both rice and *Arabidopsis* genomes (Chen *et al*., 2006). The R2R3‐type MYB domain is the minimum DNA‐binding domain, and an activation/repression domain, the balance between activators and repressors in this TFs family, may provide extra flexibility in terms of transcriptional regulation, while R1 appears to lose interaction with DNA (Ogata *et al*., 1994, 1995). However, C‐terminus regions of MYB genes are quite variable, allowing a wide range of regulation roles in different biological processes (Kranz *et al*., 1998).

Previous studies have provided extensive data revealing that the plant MYBs play central roles in an array of physiological and biological processes, such as organ growth and development (Mu *et al*., 2009; Volpe *et al*., 2013), controlling stomatal aperture (Liang *et al*., 2005), primary and secondary metabolism (Czemmel *et al*., 2009; Mellway *et al*., 2009; Wang *et al*., 2010), cold and hormonal signalling (Lee and Seo, 2015; Seo *et al*., 2009; Shim *et al*., 2013), phenylpropanoid biosynthesis (Borevitz *et al*., 2000), anthocyanin biosynthesis (Matsui *et al*., 2008; Park *et al*., 2008), signal transduction (Borevitz *et al*., 2000) and cell division (Muller *et al*., 2006; Ryu *et al*., 2005). In addition, many evidences indicate that the MYB genes are closely associated with plant responses to abiotic stresses (Agarwal *et al*., 2006; Dubos *et al*., 2010; Kim *et al*., 2013; Lee and Seo, 2015). So far, well‐characterized MYBs involved in abiotic stress response include *AtMYB1*,* AtMYB2*,* AtMYB15*,* AtMYB41*,* AtMYB44*,* AtMYB60*,* AtMYB74* and *MYB96* of *Arabidopsis* (Baek *et al*., 2013; Jung *et al*., 2008; Lee and Seo, 2015; Lippold *et al*., 2009; Oh *et al*., 2011; Seo *et al*., 2009, 2011; Wang *et al*., 2015; Xu *et al*., 2015); *OsMYB2*,* OsMYB3R‐2*,* OsMYB511* and *MYBS3* of rice (Huang *et al*., 2015a,b; Ma *et al*., 2009; Su *et al*., 2010; Yang *et al*., 2012); *GmMYB76*,* GmMYB92* and *GmMYB100GmMYB177* of soya bean (Liao *et al*., 2008; Yan *et al*., 2015).

It is conceivable that the MYBs may achieve their function in stress tolerance via regulating a variety of stress‐responsive genes, either regulatory or functional ones, which are considered as the potential target genes of the MYBs. Although a myriad of stress‐responsive genes have been tested so far, knowledge concerning the target genes of MYBs is still poorly understood. So, it is necessary to investigate how a MYB gene can affect the expression of genes involved in other metabolic pathways which have been less examined. In this study, we report the functional identification of a R2R3‐type MYB gene from *Pyrus betulaefolia*, designated as *PbrMYB21* in drought tolerance, belongs to the R2R3‐type and shares high degree of sequence similarity to *MdMYB21*. In addition, a connection between plant MYBs and ADC‐mediated polyamine accumulation has never been established although they have been separately verified as key players in abiotic stress tolerance. These results showed that *PbrMYB21* acts as a positive regulator of drought tolerance, which may be partly ascribed to its role in modulating polyamine levels through regulating arginine decarboxylase, suggesting that *ADC* might be a potential target gene of *PbrMYB21*.

## Results

### Isolation and analysis of *PbrMYB21*

**Figure 1:**
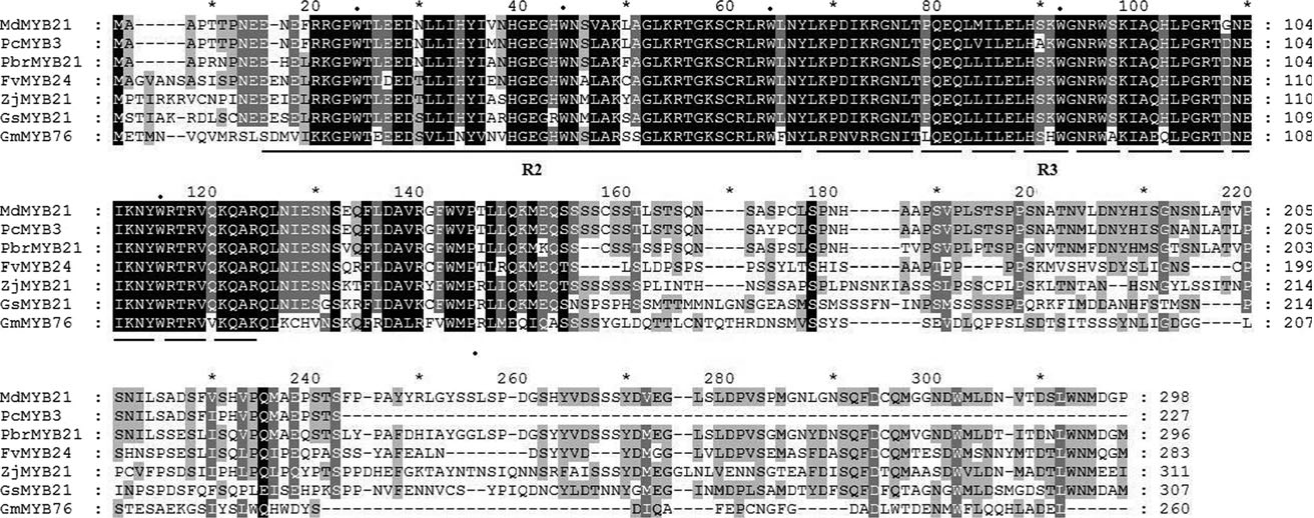
Multiple sequence alignment of PbrMYB21 and MYBs from other plant species, including *Malus domestica* (MdMYB21, NP_001280981), *Pyrus communis* (PcMYB3, AGL81356), *Fragaria vesca* subsp. *vesca* (FVMYB24, XP_004299054), *Ziziphus jujuba* (ZjMYB21, XP_015895640), *Glycine soja* (GsMYB21, KHN11407), *Glycine max* (GmMYB76, NP_001235761). The R2 and R3 domains are indicated by solid line and dotted line, respectively. Solid circles indicate the conserved residues, such as tryptophan (W).

We previously obtained a dehydration‐induced MYB TF (Pbr028812.1) from a transcriptome of *Pyrus betulaefolia* (Li *et al*., 2016). Sequence analysis demonstrated that it was a full‐length sequence with a complete open reading frame (ORF), which encodes a protein of 296 amino acid residues with a calculated molecular mass of 33.2 kDa and an isoelectric point of 5.98. Homology search showed that the gene shared the highest identity with *MdMYB21*, so it was designated as *PbrMYB21* (*Pyrus betulaefolia* MYB21). Multiple sequence alignment showed that PbrMYB21 protein had a conserved R2R3 region in its N‐terminal region, but the C‐terminal regions are divergent. The R2R3 domain contained highly conserved tryptophan residues, such as three tryptophans in the R2 repeat and two tryptophans in the R3 repeat (Figure 1). In addition, a phylogenetic tree was constructed using amino acid of PbrMYB21 and MYBs of other plants, such as *Malus domestica*,* Pyrus communis*,* Citrus sinensis* and *Glycine max*. In the phylogenetic tree, PbrMYB21 was most closely related to MdMYB21 as they shared 82% sequence identity (Figure S1).

### Expression patterns of *PbrMYB21* under various treatments

**Figure 2:**
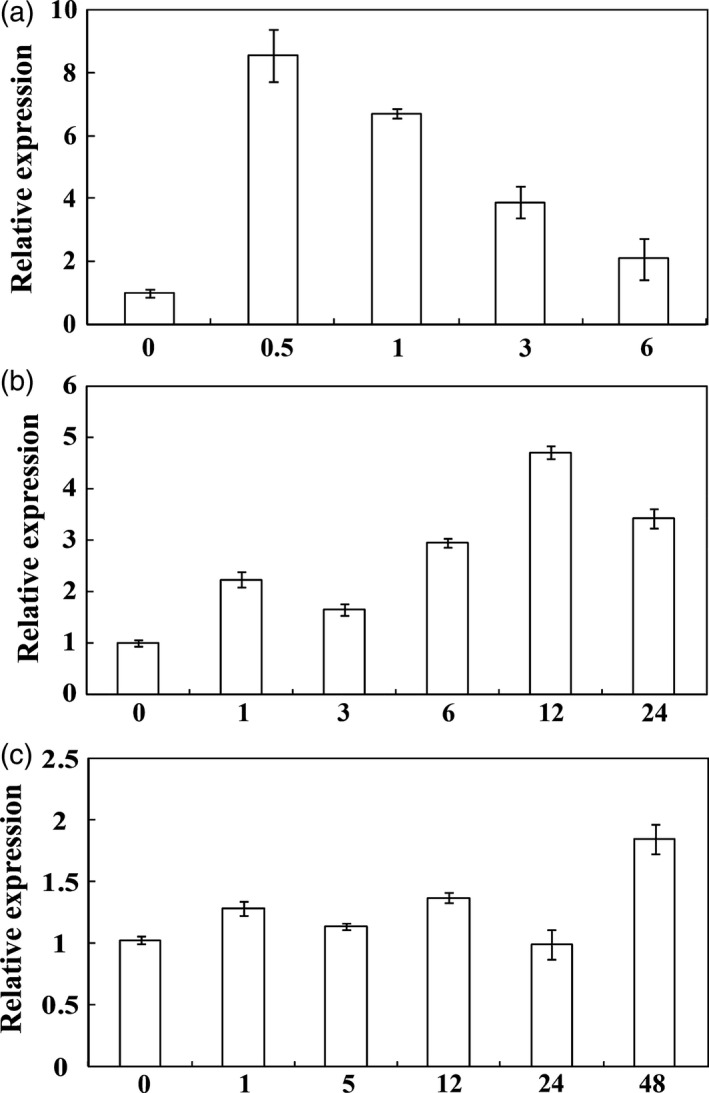
Time‐course expression levels of *PbrMYB21* in *Pyrus betulaefolia* under abiotic stresses. (a–c) expression of *PbrMYB21* in response to dehydration (a), salt (b) and cold stress (c). The samples were collected at the designated time points and analysed by qPCR. Error bars stand for SD based on four replicates.

Real‐time quantitative PCR (qPCR) was used to examine the expression profiles of *PbrMYB21* under various treatments, including dehydration, salt and cold. *PbrMYB21* mRNA was quickly and sharply accumulated within 0.5 h of dehydration, and maintained stable at 1 h, but decreased thereafter (Figure 2a). Salt treatment for 1 h up‐regulated *PbrMYB21*, which rose quickly to the peak value at 12 h, and then decreased within the 24 h (but still 3.4‐fold higher than the initial level) (Figure 2b). When subjected to cold treatment, the transcript of *PbrMYB21* did not change notably except a slight increase at 48 h (Figure 2c), indicating that *PbrMYB21* was not cold‐inducible.

### PbrMYB21 was localized to nucleus

**Figure 3:**
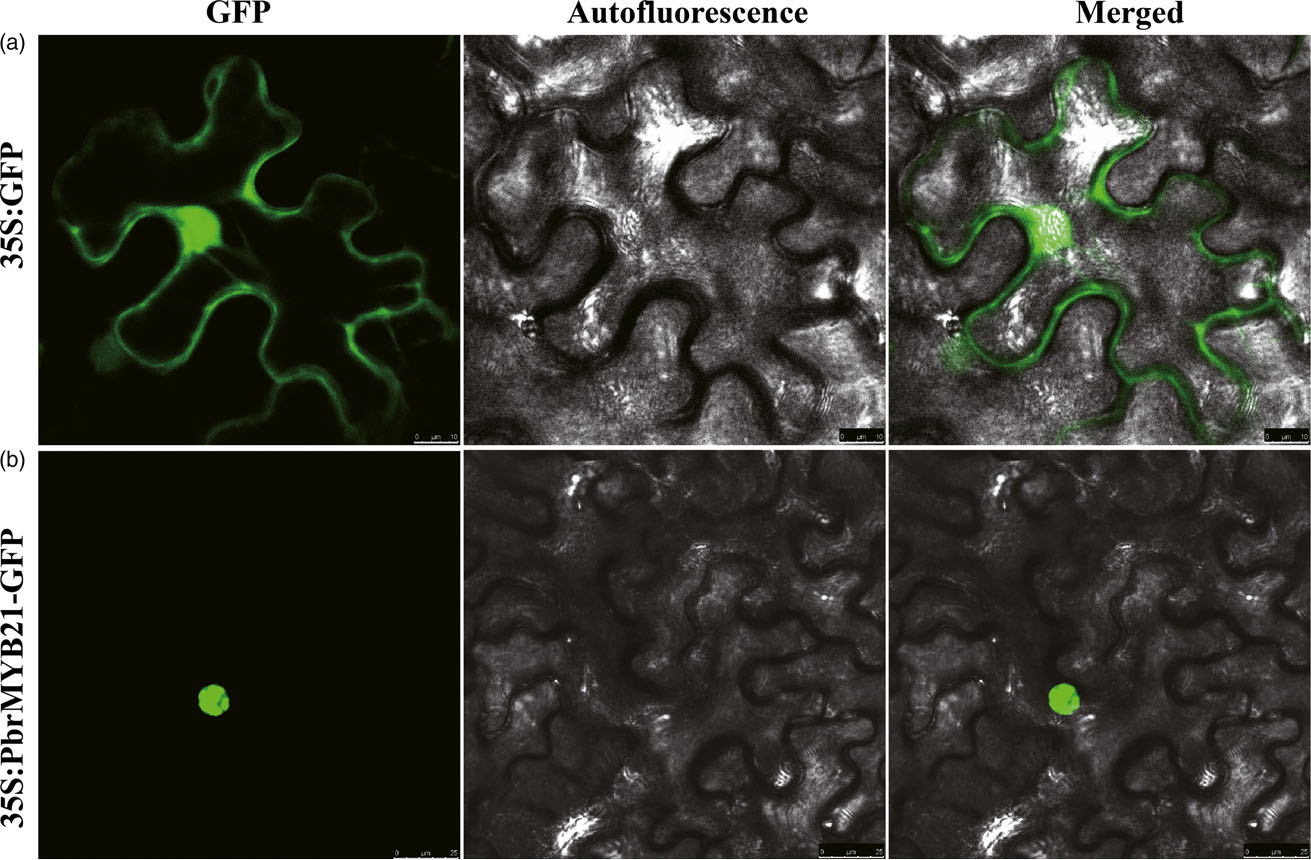
Subcellular localization of PbrMYB21. Tobacco epidermal cells were transiently transformed with constructs containing either control (GFP alone, (a) or fusion plasmid (PbrMYB21: GFP, (b)). Images under blight field (middle), fluorescence (left) and the merged images (right) are shown on the right.

To find out the subcellular localization of PbrMYB21, full‐length ORF of *PbrMYB21* was fused to N‐terminal of GFP reporter protein driven by CaMV 35S promoter, generating a fusion protein PbrMYB21: GFP, using the plasmid containing GFP alone as control. The fusion protein (PbrMYB21) and the control (GFP) were analysed in tobacco leaf epidermis via *Agrobacterium*‐mediated transformation. Microscopic visualization showed that the control GFP was uniformly distributed throughout the whole cell (Figure 3a), whereas the PbrMYB21‐GFP fusion protein was observed exclusively in the nucleus (Figure 3b). These results indicated that PbrMYB21 was a nuclear protein.

### PbrMYB21 had transactivation activity

**Figure 4:**
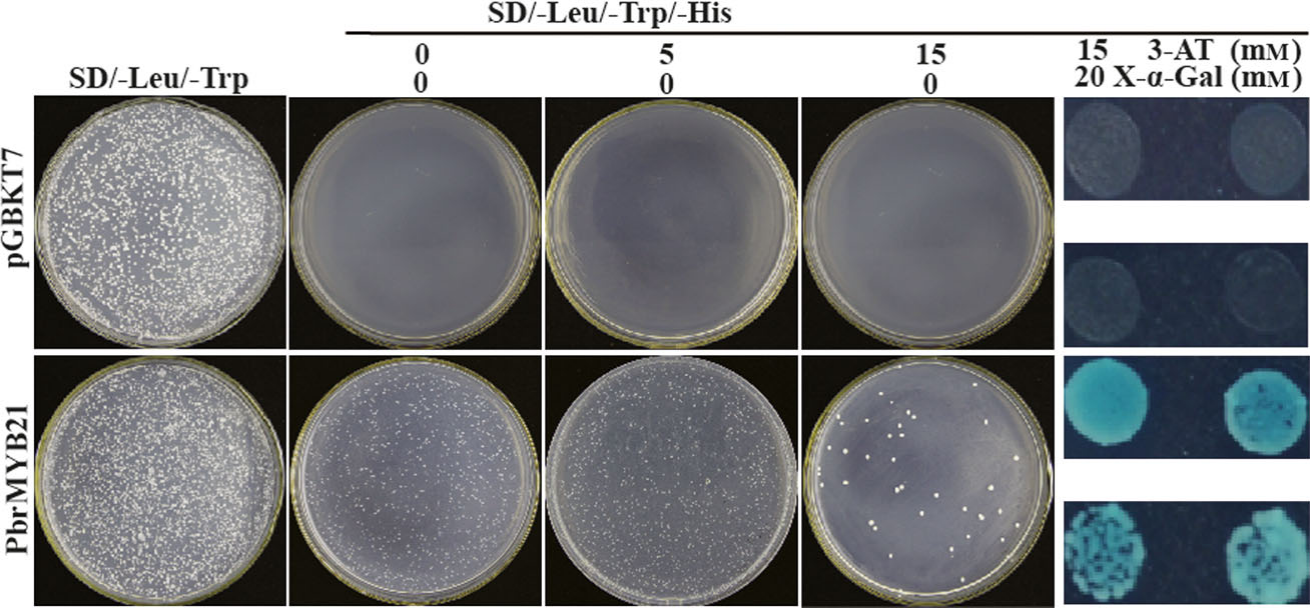
Transcriptional activation assay of PbrMYB21 in yeast. Growth of yeast cells (strain AH109) transformed with either control vector (upper panels) or fusion vector harbouring PbrMYB21 (bottom panels) on SD/–Leu/–Trp or SD/–Leu/–Trp/–His with or without 3‐AT and 20 mm X‐a‐gal.

Transactivation activity is another defining feature for a transcription factor in addition to nuclear localization. The Y2H system was used to investigate whether PbrMYB21 functioned as a transcriptional activator. For this purpose, the full‐length PbrMYB21 coding region was fused to the DNA‐binding domain of GAL4 to generate a fusion plasmid, which was transformed into yeast AH109 and growth of the cells was compared with those transformed with the control plasmid (pGBKT7) on the same selection medium synthetic dropout (SD)/‐Leu/‐Trp. The yeast cells transformed with either vector could grow on the synthetic dropout medium (SD/‐Leu/‐Trp), indicating that the analysis system was reliable. On the SD/‐Leu/‐Trp/‐His medium, the cells transformed with the control plasmid could not grow, whereas only the cells transformed with the recombinant vector survived on the selection medium alone or supplemented with 15 mM 3‐amino‐1,2,4‐triazole (3‐AT) (Figure 4, upper panels). In addition, when the yeast cells were cultured on the SD/‐Leu/‐Trp/‐His medium added with both 15 mm 3‐AT and 20 mm X‐a‐Gal, only those transformed with the fusion plasmid turned blue (Figure 4, bottom panels). Taken together, these results demonstrate PbrMYB21 had transactivation activity in yeast.

### Overexpression of *PbrMYB21* confers enhanced dehydration and drought tolerance

Given that dehydration stress resulted in the strongest induction of *PbrMYB21* transcript, we speculated that *PbrMYB21* may play an important role in drought stress response. To test whether this hypothesis is true, transgenic tobacco overexpressing *PbrMYB21* were generated by *Agrobacterium*‐mediated transformation, under the control of CAMV 35S promoter. Totally, twelve transformants (T_0_ generation) were identified as positive lines by genomic PCR analysis. Semi‐quantitative RT‐PCR analysis showed that *PbrMYB21* (GSP1, Table S1) was overexpressed in six tested lines (Figure S2), from which three independent overexpression lines of tobacco (designated as OE‐20, OE‐17 and OE‐9 hereafter) with high transcript levels of *PbrMYB21* were selected for following experiments, along with the corresponding untransformed wild type. These six overexpression lines of tobacco were further used for Western blotting assays with an anti‐His antibody (Figure S2G), consistent with the results of the semi‐quantitative RT‐PCR. Southern blotting was carried out to examine *PbrMYB21* gene copy number in the transgenic tobacco genome. As shown in Figure S3, hybridization of three transgenic lines (OE‐20, OE‐17 and OE‐9) genomic DNA digested with one restriction enzyme *Kpn*I using a 200‐bp partial fragment of *PbrMYB21* as probe produced only one visible signal under high stringency conditions. As the probe sequence did not contain any recognition sites of the *Kpn*I, the hybridization pattern suggested that the *PbrMYB21* gene exists as a single‐copy gene in the three transgenic lines genome.

**Figure 5:**
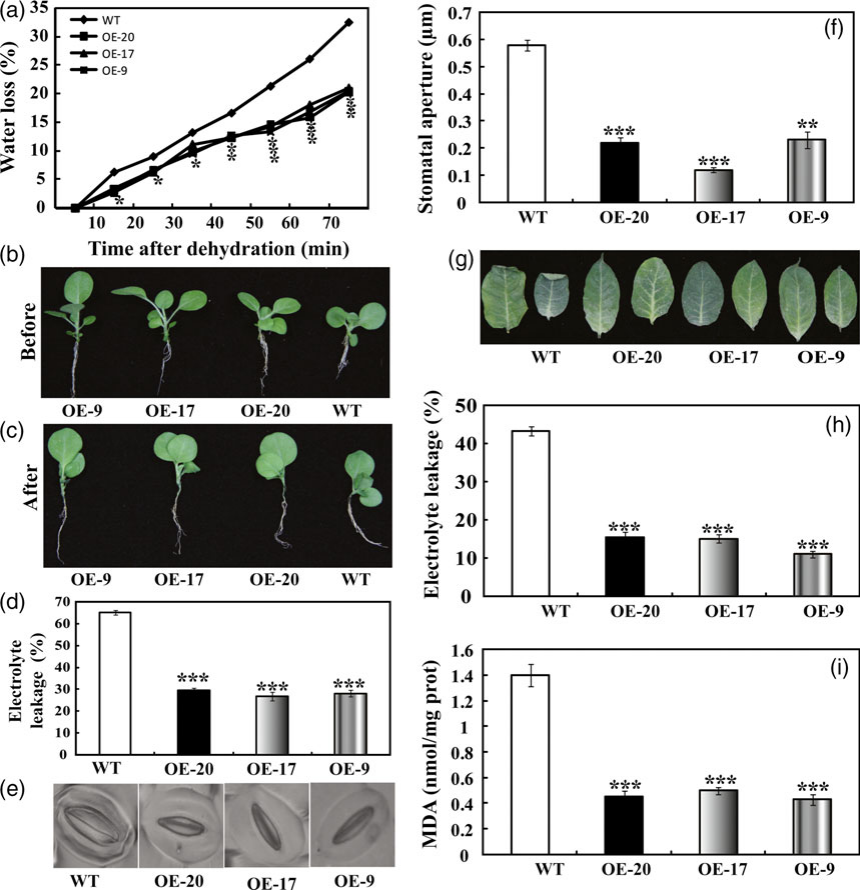
Overexpression of *PbrMYB21* conferred enhanced dehydration and drought tolerance in tobacco. (a) Time‐course fresh water loss of WT, OE‐20, OE‐17 and OE‐9 during 70‐min dehydration. (b, c) Representative images of WT, OE‐20, OE‐17 and OE‐9 after dehydration for 70 min. (d, e) Electrolyte leakage (d) and stomatal aperture size (e) of WT, OE‐20, OE‐17 and OE‐9 after dehydration for 70 min. (g) Phenotypes of 7‐week‐old plants of transgenic lines (OE‐20, OE‐17 and OE‐9) and WT after dehydration for 70 min. (h, i) Electrolyte leakage (h) and MDA contents (i) in the three lines measured after dehydration for 70 min. Asterisks indicate that the value is significantly different from that of the WT at the same time point (*P <* *0.05; **P <* *0.01; ***P <* *0.001).

When the aerial parts of 5‐week‐old *in vitro* seedlings were dehydrated in an ambient, a steady decrease in FW (fresh weight) was observed in both the WT and the three transgenic lines. However, at any time point within 70 min of dehydration, the three transgenic lines lost remarkably less water compared with the WT (Figure 5a). When dehydration was completed, leaves of WT exhibited more serious wilting relative to the transgenic lines (Figure 5b, c). EL (Electrolyte leakage), an important indicator of membrane injury, was significantly higher in the WT (65.2%) than in OE‐20 (29.7%), OE‐17 (26.9%) or OE‐9 (30.5%), indicating that the WT suffered from severe membrane damage (Figure 5d). At the end of dehydration, stomatal apertures of WT were significantly larger than those of the transgenic lines (Figure 5e, f), consistent with their water loss dynamics. The leaves of 7‐week‐old *in vitro* seedlings were also subjected to dehydration for 70 min, followed by EL measurement and MDA contents. After 70 min of dehydration, more severe leaf withering was observed in the WT plants when compared with the transgenic plants (Figure 5g). In agreement with the enhanced dehydration tolerance, the transgenic lines had lower values of EL and MDA following exposure to dehydration conditions than did the wild type (Figure 5h, i). These findings demonstrate that the transgenic lines were more resistant to dehydration.

**Figure 6:**
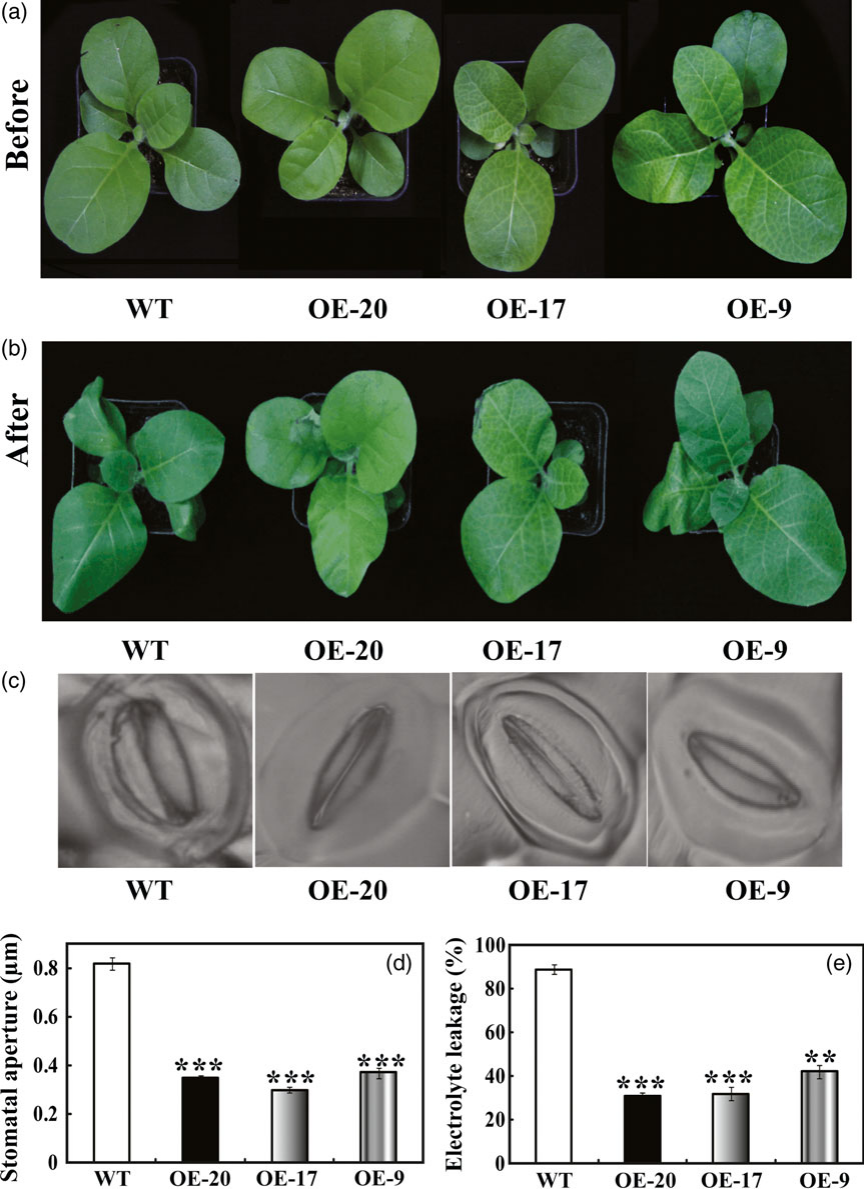
Overexpression of *PbrMYB21* conferred enhanced drought tolerance in tobacco. (a–b) Phenotypes of 7‐week‐old transgenic plants and WT before (a) and after (b) 7 days drought stress. (c–d) Representative images (c) and stomatal aperture size (d) of WT, OE‐20, OE‐17 and OE‐9 after drought. (e) Electrolyte leakage of the WT, OE‐20, OE‐17 and OE‐9 after drought treatment. Asterisks indicate that the value is significantly different from that of the WT at the same time point (**P <* *0.01; ***P <* *0.001).

Drought tolerance of the transgenic lines was also assessed using 45‐day‐old plants grown in soil pots (Figure 6a). Morphological difference became apparent after watering was stopped for 7 days, leaf wilting was more evident in the WT relative to the three transgenic lines (Figure 6b). At the end of drought, stomatal apertures of WT were significantly larger than those of the transgenic lines (Figure 6c, d). To compare the physiological differences, electrolyte leakage of OE‐20 (30.9%), OE‐17 (31.3%) and OE‐9 (42.1%) was significantly lower in comparison with 89.5% of WT (Figure 6c). These results suggest that overexpression of *PbrMYB21* in tobacco conferred pronounced enhancement of drought stress tolerance.

### Silencing of *PbrMYB21* in *Pyrus betulaefolia* confers sensitivity to drought stress

**Figure 7:**
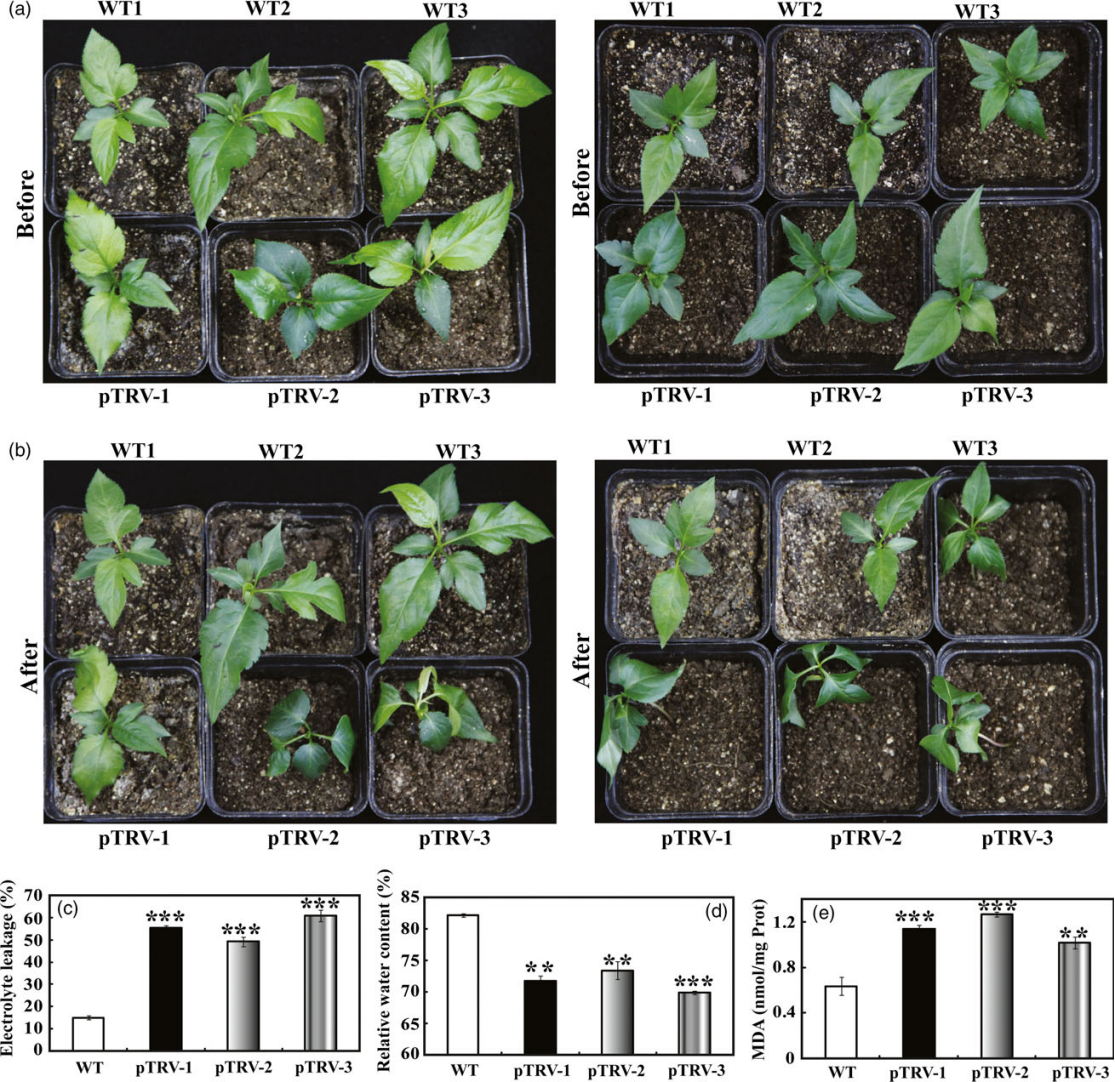
Silencing of the *PbrMYB21*i n *Pyrus betulaefolia* by virus‐induced gene silencing (VIGS) resulted in elevated drought sensitivity. (a–b) Phenotypes of *PbrMYB21‐*silenced plants before (a) and after drought stress for 7 days (b). (c–e) Electrolyte leakage (c), relative water content (d) and MDA levels (e) of the pTRV (WT) plants and pTRV‐*PbrMYB21* silencing plants (pTRV‐1, pTRV‐2 and pTRV‐3) after drought stress for 7 days. Asterisks indicate that the value is significantly different from that of the WT at the same time point (**P <* *0.01; ***P <* *0.001).

To further elucidate the role of *PbrMYB21* in drought tolerance, a virus‐induced gene silencing (VIGS) was used to suppress the expression of *PbrMYB21* in *Pyrus betulaefolia*. Transcript analysis of the leaflets revealed that the transcripts for *PbrMYB21* were suppressed in the respective silenced plants (Figure S2H). We next compared the drought tolerance of these three silenced plants grown under drought treatment. Upon exposure to drought, pTRV‐PbrMYB21 plants (pTRV‐1, pTRV‐2 and pTRV‐3) displayed more serious wilting (Figure 7a, b) and compared with the empty vector (pTRV) transformed control plants (WT). At the end of drought, electrolyte leakage of the pTRV‐PbrMYB21 plants was significantly higher than the control plants (Figure 7c). Water deprivation for 7 days, WT plants had significantly higher RWC (relative water content) relative to pTRV‐PbrMYB21 plants (Figure 7d). The control plants displayed significantly lower malondialdehyde (MDA) contents compared with the pTRV‐PbrMYB21 plants (Figure 7e).

### Transgenic lines accumulate less ROS and the silencing lines accumulate more ROS

**Figure 8:**
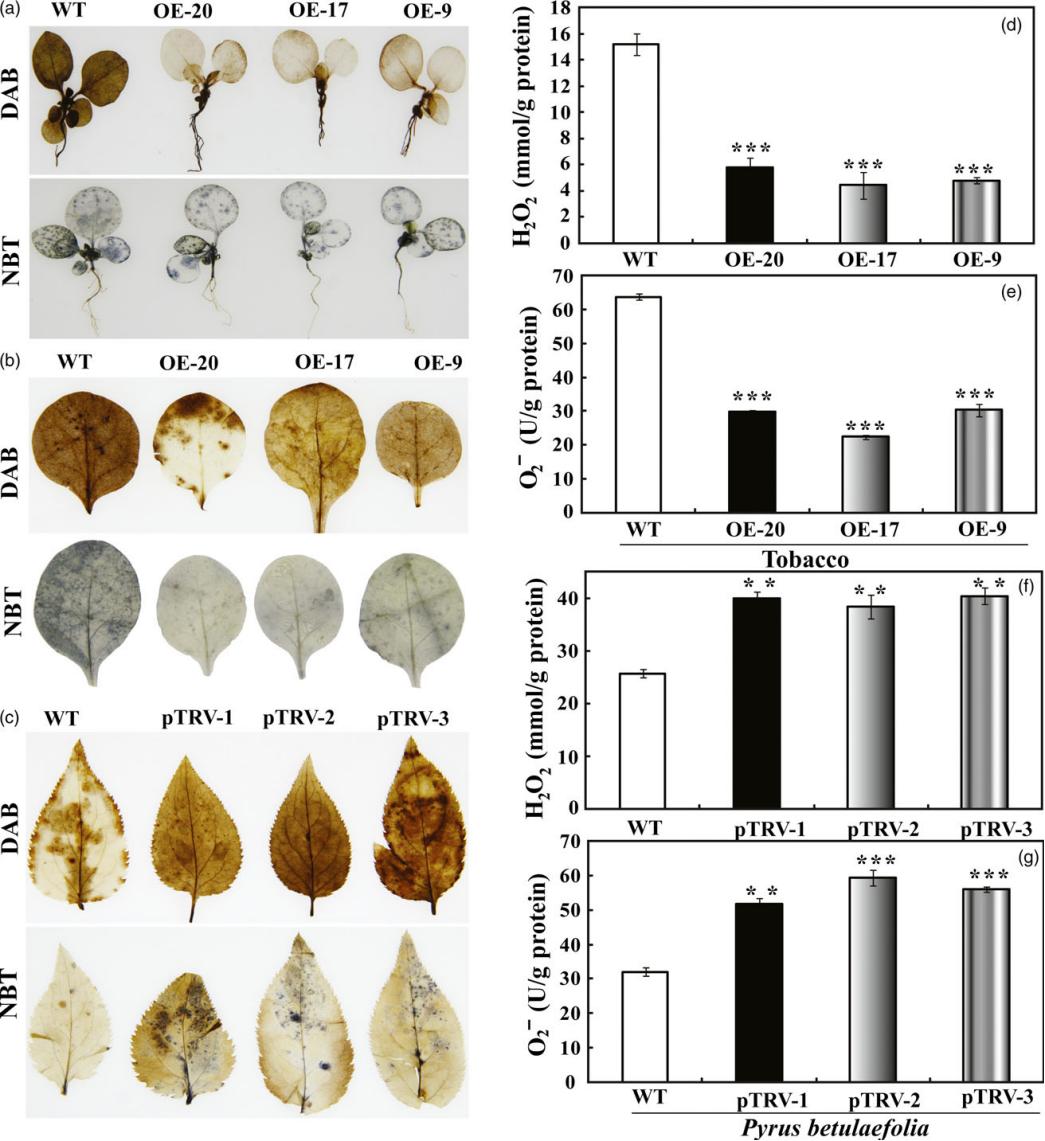
Analysis of H_2_O_2_ and anti‐O_2_^−^ in tobacco and *Pyrus betulaefolia* silenced plants after dehydration or drought stresses. (a) Histochemical staining with DAB and NBT for detection of H_2_O_2_ and O_2_^−^, respectively, in WT, OE‐20, OE‐17 and OE‐9 after 70 min of dehydration. (b) Representative images indicating *in situ* accumulation of H_2_O_2_ and O_2_^−^ in tobacco WT and transgenic lines (OE‐20, OE‐17 and OE‐9) after drought stress for 7 days. (c) Representative images indicating *in situ* accumulation of H_2_O_2_ and anti‐O_2_^−^ in *Pyrus betulaefolia* WT and pTRV‐*PbrMYB21* silencing plants (pTRV‐1, pTRV‐2 and pTRV‐3) after drought for 7 days. (d–e) Levels of H_2_O_2_ (d) and O_2_^−^ (e) in tobacco WT and transgenic lines (OE‐20, OE‐17 and OE‐9) after drought stress. The insets in (d) and (e) are histochemical staining patterns using DAB and NBT, respectively. (f–g) Levels of H_2_O_2_ (f) and O_2_^−^ (g) in *Pyrus betulaefolia* WT and pTRV‐*PbrMYB21* silencing plants after drought treatment. Asterisks indicate that the value is significantly different from that of the WT at the same time point (**P <* *0.01; ***P <* *0.001).

Histochemical staining with DAB (diaminobenzidine) and NBT (nitroblue tetrazolium) was carried out to reveal the levels of H_2_O_2_ and O_2_^−^, respectively, in the leaves subjected to dehydration and drought treatment. After the dehydration for 70 min, DAB staining showed that the intensity of brown precipitations in the WT leaves was stronger than that of the transgenic plants. Blue spots were distributed throughout the leaves of WT when NBT staining was applied, whereas leaves of the transgenic plants were only slightly stained (Figure 8a). When 45‐day‐old potted plants were subjected to drought treatment for 7 days, we also checked the ROS accumulation in the leaf discs of WT and the transgenic plants. WT leaf discs were stained to a deeper extent by DAB and NBT when compared with the transgenic counterparts (Figure 8b). Conversely, stronger DAB and NBT staining were detected in the pTRV‐PbrMYB21 plants as compared with the control plants, suggesting that the accumulation of H_2_O_2_ and O_2_^−^ was elevated when PbrMYB21 was down‐regulated (Figure 8c). To verify the histochemical staining, we quantified the levels of H_2_O_2_ and O_2_^−^ in the drought‐treated samples using a detection kit. Consistent with the histochemical staining results, quantitative measurement showed that the levels of H_2_O_2_ and O_2_^−^ in the transgenic lines were significantly lower than in WT during the drought treatment (Figure 8d, e), but the H_2_O_2_ and O_2_^−^ contents in the pTRV‐PbrMYB21 of *Pyrus betulaefolia* were significantly higher than in the WT (Figure 7f, g). Both histochemical staining and quantitative measurement indicated that the transgenic lines accumulated lower levels of ROS under the drought, whereas they were changed in an opposite way when *PbrMYB21* was silenced.

### Analysis of antioxidant enzyme activities in the transgenic plants and the silencing plants

**Figure 9:**
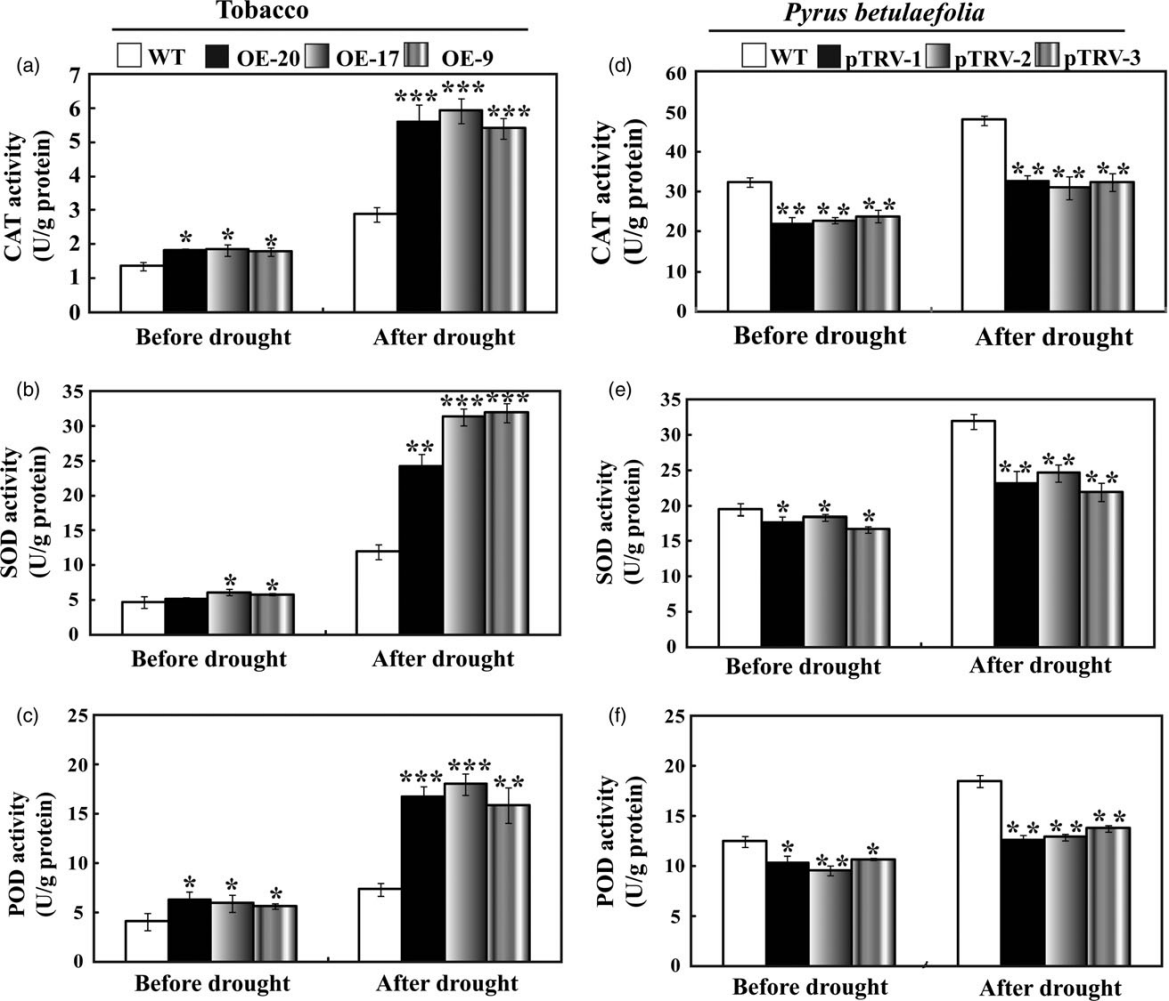
Analysis of antioxidant enzyme activities in tobacco and *Pyrus betulaefolia* silenced plants. (a–c) Activity of CAT (a), SOD (b) and POD (c) in tobacco WT and transgenic lines (OE‐20, OE‐17 and OE‐9) before and after drought treatment. (d–f) Activity of CAT (d), SOD (e) and POD (f) in *Pyrus betulaefolia* WT and pTRV‐*PbrMYB21* silencing plants (pTRV‐1, pTRV‐2 and pTRV‐3) before and after drought stress. Asterisks indicate that the value is significantly different from that of the WT at the same time point (*P < 0.05; **P < 0.01; ***P < 0.001).

The crucial role of antioxidant enzymes in ROS scavenging (Gill and Tuteja, 2010) prompted us to examine the activities of three important enzymes (SOD, CAT and POD) in WT and the transgenic lines or the silencing plants during the drought treatment. In the normal condition, the enzyme activities of SOD, POD and CAT in tobacco transgenic lines were slightly higher than those of the WT, whereas activities of the three enzymes were significantly higher in the transgenic plants compared to WT when the plants experienced drought stress (Figure 9a–c). Conversely, the three enzyme activities in the three silencing plants (pTRV‐1, pTRV‐1 and pTRV‐3) were significantly lower than those of the WT before and after drought treatment (Figure 9d–f).

### Expression analysis of stress‐responsive genes in the WT and transgenic lines or the silencing plants before and after drought stress

**Figure 10:**
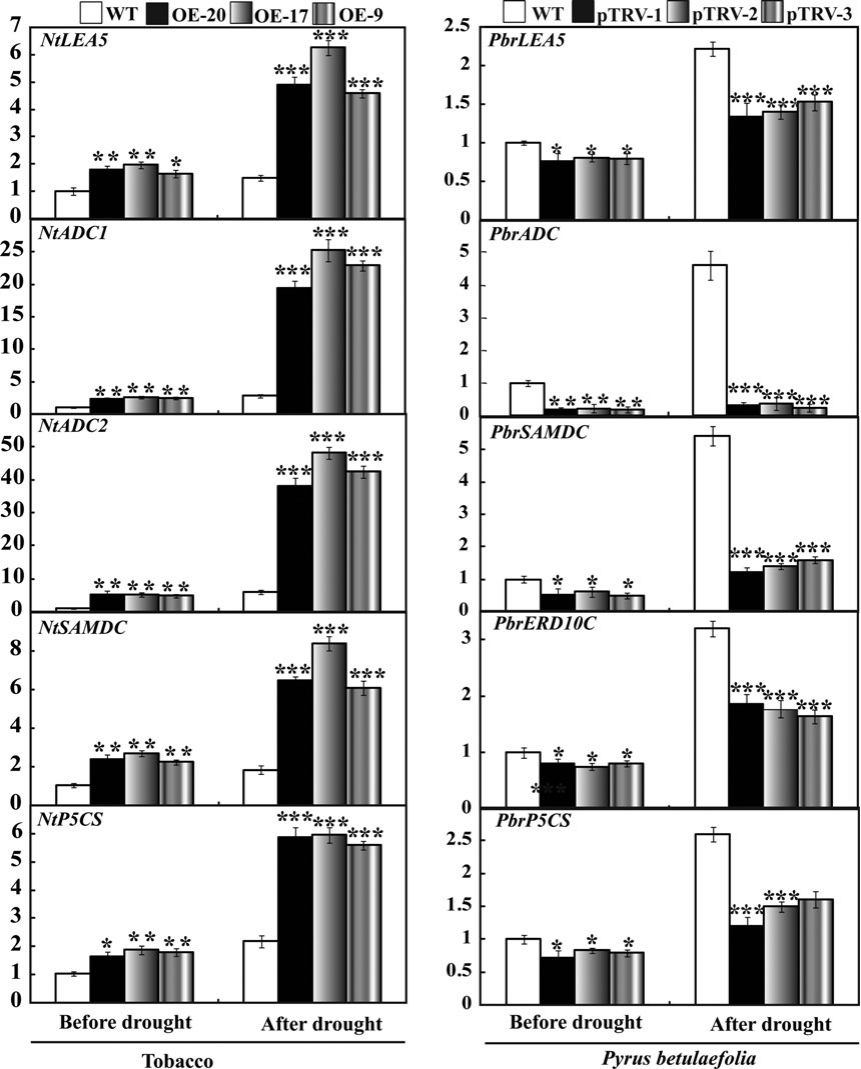
Quantitative real‐time PCR analysis of expression levels of stress‐responsive genes in tobacco and *Pyrus betulaefolia* silenced plants under normal and drought conditions. LEA5 (late embryogenesis abundant gene), ADC (arginine decarboxylase gene), SAMDC (S‐Adenosyl methionine decarboxylase), ERD10C (late embryogenesis abundant gene), *P5CS* (Δ1‐pyrroline‐5‐carboxylate synthetase). Data represent the means ± SE of four replicates. Asterisks indicate that the value is significantly different from that of the WT at the same time point (*P < 0.05; **P < 0.01; ***P < 0.001).

To elucidate the molecular mechanism underlying the enhanced drought resistance in the transgenic lines, expression patterns of some genes involved stress response, including enzymes involved in biosynthesis of polyamine (*NtADC1*,* NtADC2* and *NtSAMDC*), osmoticum adjustment or water maintenance (*NtP5CS*, and *NtLEA5*) were analysed by qRT‐PCR assay. In the absence of water, mRNA levels of all genes in three transgenic lines were significantly higher than those in the WT. Exposure to drought caused up‐regulation of the transcript levels of the examined genes in all lines, but the three transgenic lines still had a higher expression level in comparison with the WT (Figure 10). On the contrary, the mRNA abundance of these examined genes (*PbrLEA5*,* PbrADC*,* PbrSAMDC*,* PbrERD10C* and *PbrP5CS*) was prominently down‐regulated in the silencing lines before and after drought treatment. Interestingly, exposure to drought stress caused minor up‐regulation of *NtADC1* and *NtADC2* in the WT, but greater induction was observed in the transgenic lines. The transcript levels of *NtADC1* in three transgenic lines were 6.9 and 9.0 times that in the WT, respectively, while *NtADC2* levels in the three transgenic lines were 6.4–8.1 times that in the WT. The transcript levels of *PbrADC* in the silencing lines were significantly lower than in the WT without dehydration treatment. Drought increased the mRNA abundance of *PbrADC1* while the silencing lines had significantly lower levels than in WT (Figure 10). These results demonstrate that overexpression of *PbrMYB21* positively promoted the transcript level of *ADC* gene. In addition, *PtADC* of trifoliate orange has been shown previously to play a role in drought tolerance (Wang *et al*., 2011), which prompted us to focus on this protein.

### Alteration of levels of free polyamines in the transgenic plants and the silencing plants

**Figure 11:**
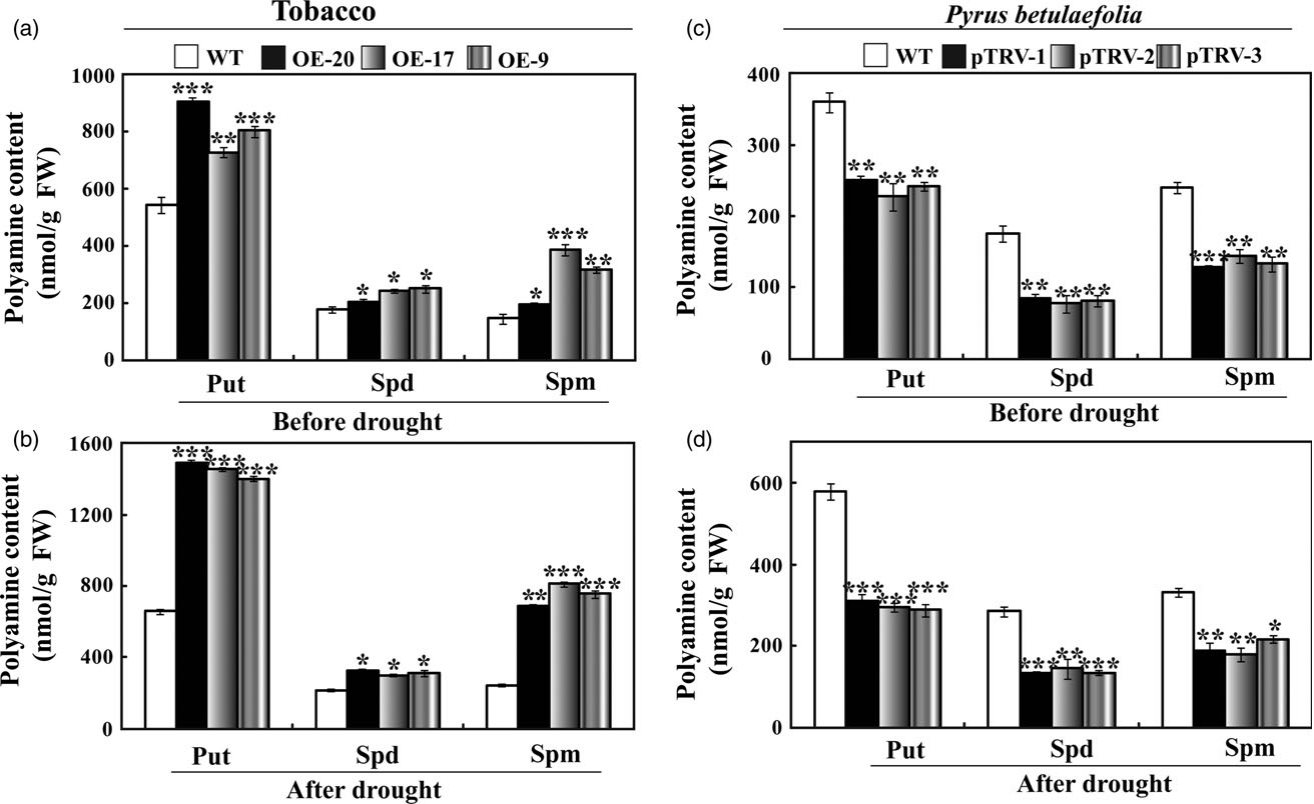
Analysis of free polyamine in tobacco and silencing *Pyrus betulaefolia* under normal and drought conditions. Free polyamine contents in WT and transgenic lines before (a) and after (b) drought stress. Put, putrescine; Spd, spermidine; Spm, spermine. (c–d) Free putrescine contents in *Pyrus betulaefolia* gene‐silenced plants and the vector control plants before (c) and after (d) drought stress. Asterisks indicate that the value is significantly different from that of the WT at the same time point (*P < 0.05; **P < 0.01; ***P < 0.001).

Further work was carried out to elucidate physiological mechanism underlying the enhanced drought tolerance rendered by *PbrMYB21*. As polyamine is an important compound involved in drought tolerance (Wang *et al*., 2011), we attempted to analyse whether polyamine synthesis was altered in the tested plants. HPLC (high‐performance liquid chromatography) was employed to analyse the free polyamine contents in the tested plants and WT. The polyamine contents in the three transgenic lines were higher than in the WT without drought stress (Figure 11a). Drought treatment led to an increase of putrescine, spermidine and spermine in all lines, whereas the elevation in the three transgenic lines was much higher than that of the WT (Figure 11b), which was consistent with the higher expression levels of *ADC* genes in the transgenic lines. Polyamine levels in the silencing plants were significantly lower than in the WT without drought treatment (Figure 11c). Drought increased the polyamine levels while the silencing plants had significantly lower levels than in WT (Figure 11d). These data demonstrate that overexpression of *PbrMYB21* elevated endogenous polyamine contents in the transgenic plants, whereas they were changed in an opposite way when *PbrMYB21* was knocked down.

### PbrMYB21 interacts with the promoters of PbrADC

**Figure 12:**
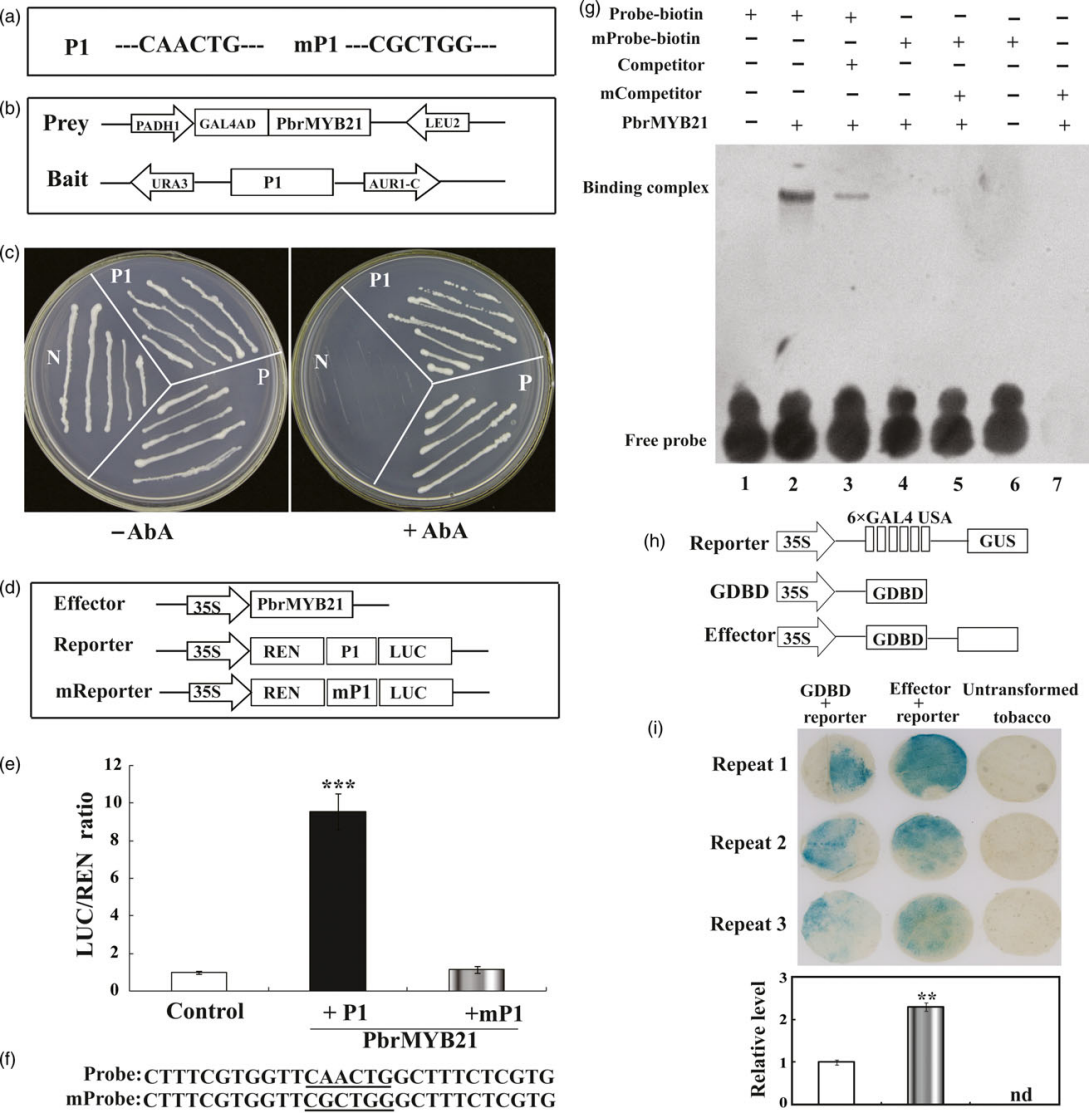
PbrMYB21 specifically binds to the promoter of *PbrADC* and acts as a transcriptional an activator. (a) Schematic diagrams of the promoter of *PbrADC*. The putative MYB binding site in the promoter fragment (P1) of *PbrADC*. Mutation of the MYB binding site sequence (mP1) is shown on the right. (b) Schematic diagrams of the prey and bait vectors used for yeast one‐hybrid assay. (c) Growth of yeast (strain Y1H Gold) cells of positive control (P), negative control (N) and bait–prey (P1) co‐transformation on SD/‐Leu medium without (left) or with (right) 300 ng/mL AbA. (d) Schematic diagrams of the effector and reporter constructs used for transient expression assay. (e) Transient expression assay of the promoter activity in tobacco protoplasts co‐transformed with effector and the reporter constructed using P1 containing normal MYB binding site element or mP1 containing mutation MYB binding site element. (f) Interaction of the PbrMYB21 protein with labelled DNA probes for the cis‐elements of the *PbrADC* promoter in the EMSA. (g) The His‐PbrMYB21 protein was incubated with the biotin‐labelled promoter fragment containing the wild‐type CAACTG or the mutated CGCCTG form; the nonlabelled fragment was used as a competitor; −, absence; +, presence. (h) Schematic diagrams of the three vectors used for the transient expression assay of the transcriptional activity of PbrMYB21 in *N. benthamiana* leaves using a GAL4/UAS‐based system. 35S, the 35S promoter without the TATA‐box; 6 × GAL4 UAS, six copies of the GAL4‐binding site; G4DBD, the GAL4 DNA‐binding domain; effector, PbrMYB21 was inserted downstream of GDBD. (i) GUS staining (top) and relative expression level (bottom) of the *N. benthamiana* leaves co‐transformed with the indicated plasmids. Untransformed *N. benthamiana* leaves were used to show the original colour. The asterisks indicate a value that is significantly different (**P < 0.01). nd, Not detected. Experiments were performed three times, and each experiment contained at least three replicates.

The elevation or decrease of ADC genes in *PbrMYB21*‐overexpressing or silencing plants, respectively, implied that ADC gene might be one of the potential target genes that are regulated by PbrMYB21. To verify this hypothesis, a 1980‐bp promoter of *PbrADC* was isolated by genomic PCR. The putative transcriptional start site and the TATA‐box were located 57 and 85–88 bp upstream of the translation start codon. Bioinformatics prediction demonstrated that the promoter of *PbrADC* contained putative a MYB‐recognizing motif (CAACTG, ‐673 to ‐668 bp; Figure 12a). Therefore, we investigated whether PbrMYB21 could bind to this element using a yeast one‐hybrid assay. A 246‐bp fragment containing the MYB‐recognizing element (P1) was used as bait and cloned into the reporter vector, while PbrMYB21 was used a prey. The yeast cells of positive and negative control and those co‐transformed with effector and reporter grew well on the SD/‐Ura/‐Leu medium without the antibiotic (AbA). However, when 300 μm AbA was added, growth of negative control was fully inhibited, while the yeast cells of positive control and bait–prey survived (Figure 12c). These results indicate that PbrMYB21 interacted with the promoter of PbrADC in yeast. To confirm the results of the yeast one‐hybrid assay, transient expression assay was further performed to confirm the interaction between PbrMYB21 and the promoter of PbrADC. The promoter activities, expressed as LUC/REN ratio, of the PbrMYB21 were significantly higher than those in the WT (Figure 12d, e). In contrast, when MYB *cis*‐element in the promoter of PbrADC was mutated, there was no activity in the samples transformed with vectors of the negative control and mutated MYB element.

To further determine whether PbrMYB21 specifically binds to the MYB recognition site in the PbrADC promoter, electrophoretic mobility shift assay (EMSA) was performed using prokaryon‐expressed and purified PbrMYB21‐His fusion proteins. A specific DNA‐PbrMYB21 protein complex was detected when the CAACTG‐containing oligonucleotide was used as a labelled probe, and the same oligonucleotide that was unlabelled was used as a competitor (Figure 12f). The formation of these complexes was reduced with increasing amounts of the unlabelled competitor probe with the same sequence. In addition, when the cis‐acting element was mutated from CAACTG to CGCTGG, a protein–DNA complex was not detected in the presence of the PbrMYB21 protein, indicating that the binding was specific for the CAACTG sequence (Figure 12g). The specificity of this competition confirms that PbrMYB21 recognized and bound specifically to the CAACTG motif within the PbrADC promoter.

The results above demonstrate that PbrMYB21 has transcriptional activity, but it remains to be verified whether it is an activator or a repressor. To address this, we performed a classical GAL4/UAS‐based system assay to study the transcriptional activation or repression of a protein (Wang *et al*., 2014). As shown in Figure 12h, the GAL4 DNA‐binding domain (G4DBD) that binds to the six copies of UAS to activate GUS expression. We then generated a fusion protein, G4DBD‐PbrMYB21, which was co‐transformed with 35S‐UAS‐GUS in *N. benthamiana* leaves to determine PbrMYB21 transcriptional activity. Histochemical staining showed that GUS expression was prominently activated in the *N. benthamiana* leaves compared with the leaves transformed with the empty vector control (Figure 12i). These results suggest that PbrMYB21 might act as a transcriptional activator of *PbrADC*.

## Discussion

The MYB proteins constitute one of the largest transcription factor families in plants; 118 and 222 MYBs have been unravelled in the genomes of grape and apple. Numerous studies have provided extensive data revealing that plant MYB proteins play central roles in an array of physiological and biological processes, such as primary and secondary metabolism, cell cycle progression, hormonal signalling, and cell patterning and tissue differentiation, stomatal movement, signal transduction and cell division (Cominelli *et al*., 2005; Li *et al*., 2013; Mellway *et al*., 2009; Mu *et al*., 2009; Ryu *et al*., 2005; Shim *et al*., 2013). In addition, accumulating evidence shows that MYB proteins play pivotal roles in plant responses to various abiotic stresses, including salt (Guo *et al*., 2016; Wang *et al*., 2015), drought (Guo *et al*., 2013), cold (Cominelli *et al*., 2005) and phosphate starvation (Baek *et al*., 2013). Although some MYB TFs have been characterized, the biological functions of most of the plant MYB TFs remain unclear, particularly in nonmodel plants, they are still poorly understood. Therefore, elucidation of the functions of MYBs in perennial plants, such as *Pyrus betulaefolia*, will advance understanding on the roles of MYBs. Of special note, the target genes in the identified MYBs, even those well‐characterized ones, are still not clearly dissected so far. Therefore, target gene exploration is still one major challenge to gain a more comprehensive understanding on the roles and modes of action underlying the stress tolerance of the MYB genes.

MYB‐containing genes have greatly diversified, being classified into four major subfamilies, namely 1R‐MYB proteins, R2R3‐MYB proteins, 3R‐MYB proteins, and 4R‐MYB proteins, containing one, two, three or four MYB domain repeats, respectively. R1R2R3‐MYB proteins are commonly found in animals, but the majority of plant MYB genes belong to the R2R3‐MYB types. In this study, we isolated a R2R3‐type MYB from *Pyrus betulaefolia*. PbrMYB21 has the entire set of above‐mentioned signature motifs required for defining a typical R2R3‐type MYB, despite a low degree of sequence conservation outside the MYB DNA‐binding domain. Multiple alignments revealed that PbrMYB21 shared a striking sequence similarity with MdMYB21 of *Malus domestica* and other plants, indicating that *PbrMYB21* is a putative MYB homologue of *Pyrus betulaefolia*.

The transcript levels of *PbrMYB21* were increased by dehydration and salt, in particular the former, indicating that *PbrMYB21* may be involved in the signal transduction of these two types of stresses. Expression patterns of *PbrMYB21* were largely similar to *AtMYB41* that has been shown to be strongly and continuously induced by salt and drought (Lippold *et al*., 2009). However, it has to be mentioned that *PbrMYB21* was not induced by abscisic acid treatment, and they also different from *AtMYB41*, as *PbrMYB21* was not induced under abscisic acid. Compared with salt stress, dehydration caused higher induction of *PbrMYB21* transcript levels, which forced us to do in‐depth work and elucidate its function in drought tolerance. The three selected transgenic lines overexpressing *PbrMYB21* displayed better tolerance to dehydration and drought, while knockdown of *PbrMYB21* rendered the silencing plants more susceptible to drought, indicating that *PbrMYB21* acted as positive regulator of drought tolerance.

The overexpressing plants of tobacco displayed enhanced dehydration tolerance, as indicated by slower water loss rates and lower levels of MDA than the WT. As lipid peroxidation, represented by MDA levels and ELs, is detrimental to the membrane systems, it is conceivable that the transgenic plants are suffered from lower degree of membrane injury under the drought stress when compared with the WT. Lipid peroxidation largely results from oxidative stresses due to the excessive accumulation of ROS, in particular H_2_O_2_ and O_2_^−^. Histochemical staining by DAB and NBT clearly demonstrated that under drought stress the three transgenic lines accumulated remarkably less O_2_^−^ and H_2_O_2_ than WT, implying they experienced milder oxidative stresses relative to the WT, consistent with the data on MDA and EL. As ROS accumulation under stressful situations largely relies on the homoeostasis between ROS generation and removal, accumulation of less ROS in the transgenic lines seems to indicate that scavenging systems in these plants might work more effectively compared with WT. To detoxify stress‐induced ROS, plants have evolved a set of ROS detoxification system, in which several enzymes play essential roles, maintaining the ROS homoeostasis at a favourable situation so that the cells are less influence by the oxidative stress. ROS detoxification in higher plants is mainly achieved by ROS‐scavenging enzymes, such as SOD, CAT and POD (Miller *et al*., 2010). In this work, transgenic plants had higher activities of three antioxidant enzymes (CAT, POD and SOD), implying that they possess a more efficient enzymatic antioxidant system to eliminate ROS produced compared with the WT, which is consistent with the dramatic reduction of ROS level and ROS‐associated membrane damage (lower MDA and EL). In this regard, it is seemingly likely that overexpression of *PbrMYB21* in the transgenic plants can alleviate ROS accumulation in a more powerful way. In contrast, the *PbrMYB21*‐silenced *Pyrus betulaefolia* plants exhibited larger electrolyte leakage, higher MDA levels, higher H_2_O_2_ and O_2_^−^ levels, and lower enzymes activities, indicating that suppression of PbrMYB21 rendered the silencing plants more susceptible to the drought stress. The plant phenotype or parameters of the silencing plants were opposite to what was seen with the overexpression lines.

To cope with unfavourable environmental constraints, plants modulate the expression of a large scale of stress‐responsive genes, constituting an important molecular basis for the response and adaptation of plants to stresses (Umezawa *et al*., 2006). To gain a deeper understanding of the regulatory function of *PbrMYB21* and to explain the enhanced the drought tolerance at molecular levels, transcript levels of some stress‐responsive genes were monitored before and after drought treatment, including enzymes involved in biosynthesis of polyamine (*NtADC1*,* NtADC2* and *NtSAMDC*), osmoticum adjustment or water maintenance (*NtP5CS*,* NtLEA5*), which or whose homologues in other plants have been shown to be involved in abiotic stress response. QRT‐PCR analysis showed that the expression level of these genes was higher in the transgenic plants compared with those of WT under drought stress, consistent with earlier reports in which overexpression of a TF may active a group of target genes that function in a concerted manner to counteract the adverse effects of abiotic stresses (Huang *et al*., 2013). Although expression levels of all of the tested genes were up‐regulated by drought, they were still higher in the transgenic plants than in WT, indicating that these genes were more intensely induced in the transgenic lines. Interestingly, two genes (*NtADC1* and *NtADC1*) involved in polyamine synthesis displayed significantly higher mRNA abundance compared with the other stress‐related genes. In additional, we also found that suppression of *PbrMYB21* in the silencing plants was accompanied by a noticeable reduction of these stress‐responsive genes, particularly *PbrADC*, was lower in the silencing plants than in WT before and after drought treatment. *LEA5* and *ERD10C* encode hydrophilic late embryogenesis abundant (LEA) proteins that are assumed to play critical roles in combating cellular dehydration tolerance by binding water, and protecting cellular and membrane damage (Hundertmark and Hincha, 2008). *P5CS*, a key enzyme of proline biosynthesis, the transcript levels of this gene of the transgenic plants were higher than that of the WT before and after drought stress. *ADC1* and *ADC2* are genes involved in biosynthesis of polyamines, which are low molecular weight polycations and have been shown to be important stress molecules (Liu *et al*., 2007). A number of earlier studies showed that more drastic induction of these genes implied that the transgenic plants might synthesize higher levels of polyamines to prevent them from lethal injury and maintain better growth under water stress (Shi *et al*., 2010). In this study, we showed that increased *PbrMYB21* expression led to higher polyamines accumulation, which might contribute to the improved dehydration or drought in the transgenic plants on the basis of the following two arguments. Firstly, higher levels of *ADC* transcripts and free polyamines were detected in the transgenic lines, concomitant with accumulation of less ROS, which was positively consistent with their stress tolerance capacity. Secondly, *PbrMYB21*‐silenced decreased the transcript level of *ADC* gene and polyamine contents and resulted in substantial impairment of drought tolerance. All of these results demonstrate that promotion of polyamine accumulation constituted one of the physiological or metabolic bases responsible for the enhanced drought tolerance in the transgenic plants overexpressing *PbrMYB21*.

Taken together, we isolated a R2R3‐type MYB from *Pyrus betulaefolia*, which acts as a positive regulator of drought tolerance. The higher levels of *ADC* transcripts and free polyamines were detected in the *PbrMYB21* transgenic lines, whereas VIGS of *PbrMYB21* in *Pyrus betulaefolia* down‐regulated *ADC* abundance and decreased free polyamine level, in agreement with these two genes common up‐regulated from *Pyrus betulaefolia* drought transcriptome data set. Bioinformatics prediction revealed the presence of one MYBR element on the promoter of *PbrADC* gene, which were shown to be interacted by *PbrMYB21*, indicating that *ADC* might serve as a direct target of *PbrMYB21*. Y1H, transient expression assays and EMSA provided evidence further supporting a direct and specific interaction between *PbrMYB21* and the *PbrADC* promoter. In summary, these results indicated that *PbrMYB21* functioned in mediating drought tolerance by elevation of PAs levels via regulating *ADC* gene, which may explain the higher levels of *NtADC1*,* NtADC2* and free polyamines in the transgenic plants, concomitant with overexpression of *PbrMYB21* elevated *ADC* expression levels and polyamine content, whereas they were changed in an opposite way when *PbrMYB21* was silenced. Establishment of the MYB‐ADC network provides new valuable knowledge of the function and underlying mechanism of MYB and expands our understanding of the complex drought signalling network. However, it has to be pointed out that *PbrMYB21* also may regulate other stress‐responsive genes, extra efforts are required in the future to unravel other components related to *PbrMYB21* so as to gain a clear‐cut silhouette of the major hub in the network.

## Materials and methods

### Plant materials and stress treatment

The leaves were obtained from 45‐day‐old *Pyrus betulaefolia* seedlings which grown at Nanjing Agricultural University for analysis of expression patterns of *PbrMYB21* under various stresses. The shoots were washed and cultured for 2 days in water in a growth chamber (25 °C) to minimize the mechanical stress on the tissues, followed by exposure to corresponding stress treatments, which were carried out as follows. The seedlings were used for gene cloning and analysis of expression patterns under stresses. For cold stress, the seedlings were placed in the chamber set at 4 °C for 0, 1, 5, 12, 24 and 48 h. For dehydration treatment, the seedlings were placed on dry filter papers for 0, 0.5, 1, 3 and 6 h under ambient environment. For salt stress, the seedlings were treated with 200 mM NaCl solution for 0, 1, 3, 6, 12 and 24 h. For each treatment, at least 60 seedlings were used, and the leaves were sampled from three randomly collected seedlings at designated time point and frozen immediately in liquid nitrogen and stored at −80 °C for further analysis.

### RNA extraction and quantitative real‐time PCR analysis

Total RNA was extracted from frozen leaves using RNAiso Plus RNA (TaKaRa, China) and treated with RNase‐free DNase I (Thermo, USA) to eliminate the potentially contaminating DNA. First‐strand cDNA was synthesized using RevertAid Reverse transcriptase (Thermo, USA) according to manufacturer's instructions. Quantitative real‐time PCR was carried out with SYBR‐Green PCR kit (TakaRa, China) using 20 μL of reaction mixture consisting 10 μL of 2× SYBR‐PreMix EX Taq, 50 ng of cDNA, and 0.25 μm of each primer (GSP1, Table S1). The program of the qPCR was as follows: denaturation at 94 °C for 5 min, followed by 40 cycles of 94 °C for 10 s, 60 °C for 30 s, 72 °C for 30 s and a final extension 3 min at 72 °C. Each sample was analysed in four replicates, and the 2^−ΔΔCt^ method (Livak and Schmittgen, 2001) was applied to calculate the relative expression levels of each gene. *Tubulin* and *Ubiquitin* were analysed in parallel as reference controls for *Pyrus betulaefolia* and tobacco, respectively, to normalize expression levels.

### Gene isolation and bioinformatics analysis

In an earlier study, a dehydration‐induced MYB TF (Pbr028812.1) showing high sequence homology to MYB21 was found to be up‐regulated in the transcriptome. RNA extraction and cDNA synthesis of the relevant samples were carried out as elaborated above. Based on the above sequence, a pair of gene‐specific primers (GSP2, Table S1) was used for RT‐PCR to amplify *PbrMYB21*. The RT‐PCR, in a total volume of 50 μL, consists of 250 ng of cDNA, 1× TransStart FastPfu buffer, 0.25 mM dNTP, 2.5 U of TransStart FastPfu DNA polymerase (TRANS), 0.4 μm of each primer and nuclease‐free water. PCR was performed in a thermocycler with a programme consisting of 2 min at 95 °C, followed by 40 cycles of 20 s at 95 °C, 20 s at 55 °C, 60 s at 72 °C and a 5‐min extension at 72 °C. The PCR product was purified, subcloned into pMD18‐T vector (Takara, China) and sequenced in Invitrogen (Shanghai, China). The multiple alignments of the amino acid sequence in different species were used by ClustalW and GeneDoc software, and the phylogenetic tree was constructed by the maximum‐likelihood (ML) method in the MEGA 6.0 program, theoretical isoelectric point (*p*I) and molecular weight were predicted by Expert Protein Analysis System (http://web.expasy.org/compute_pi/).

### Transactivitional activity and subcellular localization of PbrMYB21

For the transactivation assay, the complete ORF of *PbrMYB21* was amplified by PCR using primers (GSP3, Table S1) containing *BamH*I and *Nco*I restriction sites, and the amplicon was double digested by *BamH*I and *Nco*I. The resultant fragment containing PbrMYB21 was then fused downstream of the yeast GAL4 DNA‐binding domain in pGBKT7 by recombination reactions. The fusion vector and the negative control (pGBKT7) were independently expressed in yeast strain AH109 according to the manufacturer's instructions.

The full‐length cDNA of the *PbrMYB21* was PCR amplified using primers (GSP4, Table S1) containing *Bgl*II and *Spe*I restriction sites and inserted into a polylinker site of the binary vector pCAMBIA1302 to generate a fusion construct (p35S‐PbrMYB21‐GFP). After identified the sequence, the fusion construct (p35S‐PbrMYB21‐GFP) and the control vector (pCAMBIA1302) were transferred into *Agrobacterium tumefaciens* strain GV3101 by heat shock. The abaxial surfaces of 5‐week‐old *N. benthamiana* leaves were agroinfiltration (Kumar and Kirti, 2010) with the bacterial suspension (OD600 = 0.5) and then kept in an incubator for 48 h, followed by live cell imaging under an inverted confocal microscopy (Zeiss LSM 780, Germany).

### Vector construction and plant transformation

The full‐length coding region of *PbrMYB21* was PCR amplified with GSP5 (Table S1) containing a His‐tag (HHHHHH) and inserted into a polylinker site of the binary vector pCAMBIA1301. The tobacco rattle virus (TRV)‐based vectors (pTRV1/2) construct was used for the VIGS (Wang *et al*., 2016; Xie *et al*., 2012). For the construction of pTRV‐PbrMYB21, a fragment of the PbrMYB21 ORF (292, 91–382 bp) was amplified by PCR using primers of GSP6, which was then introduced into the pTRV2 vector. Empty pTRV2 vector was used as a control. All recombinant vectors were introduced into *Agrobacterium tumefaciens* strain GV3101 by the freeze–thaw method. The overexpression vector was used to transform tobacco (*Nicotiana tabacum*), and VIGS was performed by infiltration into the leaves of 30‐day‐old *Pyrus betulaefolia* seedling with *A. tumefaciens* harbouring a mixture of pTRV1 and pTRV2‐target gene in a 1 : 1 ration (Ramegowda *et al*., 2013; Wang *et al*., 2016). Tobacco transformation was performed based on a leaf disc method (Horsch *et al*., 1985). The hygromycin‐resistant overexpression plants were PCR verified. Transcript levels of PbrMYB21 in the positive transgenic plants were analysed by RT‐PCR. Semi‐quantitative RT‐PCR was also performed to determine the gene‐silencing efficiency. T2 homozygous plants of tobacco transgenic were used for the subsequent experiments. The reference genes, *Tublin* and *Ubiquitin*, were used as internal controls for *Pyrus betulaefolia* and tobacco, respectively.

### Stress tolerance assay of the transgenic plants

Seeds of T_2_‐generation transgenic plants and wild type were sterilized for 30 s in 70% (v/v) ethanol and incubated in 2.5% (v/v) H_2_O_2_ for 5 min, followed by rinse with sterile distilled water for three times before they were sown on germination medium (GM) containing MS salts, 30 g/L sucrose and 0.8% agar (pH 5.7). For dehydration stress, two sets of experiments were designed. First, 30‐day‐old *in vitro* seedlings of the three lines removed from germination medium as mentioned above were allowed to dry for up to 70 min on the filter papers. The fresh weight (FW) of the seedlings was measured every 10 min using a scale, and the percentage FW loss was determined relative to the initial weight. For drought experiment, 5‐day‐old transgenic tobacco lines and WT were first transplanted into plastic containers filled with a mixture of soil and sand (1 : 1) where they were regularly watered for 7‐week‐old until the drought treatment. At the onset of and after 7 days of withholding water, morphological changes of the drought plants were inspected during the drought treatment. Photographs were taken when the differences were noticeable. The leaves were collected from some plants for analysis of antioxidant enzyme activity, relative water content (RWC), metabolite levels and expression of stress‐responsive genes.

### Physiological measurement and histochemical staining

MDA, H_2_O_2_ and O_2_^−^ were measured using specific detection kits following the manufacturer's instructions (Nanjing Jiancheng Bioengineering Institute, Jiangsu, China). Histochemical staining with DAB and NBT was used to analyse the *in situ* accumulation of H_2_O_2_ and O_2_^−^ according to Huang *et al*. (2011). Antioxidant enzyme (POD, SOD and CAT) activity, H_2_O_2_ level and antisuperoxide anion activity were quantified using the relevant detection kits (Nanjing Jiancheng Bioengineering Institute) based on the manufacturer's instructions. For microscopic observation of stomata, the epidermis was stripped and examined under an Olympus BH‐2 microscope (Olympus, Tokyo, Japan) equipped with an Olympus DP70CCD camera (Olympus, Tokyo, Japan). The pictures were captured as JPEG digital files, and stomatal apertures were measured using Image Pro Plus 5.0.

### Quantification of free polyamine levels by high‐performance liquid chromatography (HPLC)

Free PAs were extracted and derived according to Liu *et al*. (2009) and Shi *et al*. (2010) with slight modification. In brief, nearly 0.1 g of sample powder was extracted in 1 mL of 5% cold perchloric acid on ice for 30 min. After centrifugation at 10 800*g* (4 °C) for 15 min, the supernatant was shifted to a new tube, and the pellet was extracted in 1 mL of 5% perchloric acid again. The supernatant from two centrifugations was mixed, and 200 μL of mixture was derived with 400 μL dansyl chloride (10 mg/mL in acetone) plus 200 μL saturated sodium bicarbonate; meanwhile, 10 μL 1, 6‐hexanediamine (100 μm stock) was added as an internal standard. The derivatization was performed at 60 °C for 60 min, and then 100 μL proline (100 mg/mL) was added and the mixture was incubated for an additional 30 min at 60 °C. The dansylated polyamines were measured using a high‐performance liquid chromatography (Waters, USA) equipped with a C^18^ reversed‐phase column (4.6 mm × 150 mm, particle size 5 μm) and a fluorescence spectrophotometer with excitation and emission wavelength of 365 and 510 nm, respectively. Standard substances of PUT, SPD and SPM (sigma) were used to determine the retention times of three polyamines in the HPLC chromatograms. Three replicates were performed for each treatment.

### Yeast one‐hybrid assays in yeast

Putative MYB *cis*‐element was identified in the promoter region of the ADC gene (Pbr022368.1) based on the pear genome sequence (http://peargenome.njau.edu.cn/). Yeast one‐hybrid assay was performed to investigate whether PbrMYB21 could interact with the MYB *cis*‐element. The full‐length ORF of *PbrMYB21* was amplified by PCR using primers (GSP7, Table S1) and integrated into the *BamH*I and *Nco*I sites of pGADT7‐Rec (Clontech) to create a prey vector (pGADT7‐PbrMYB21). Based on the distribution of the MYB *cis*‐element, one fragment (P1, ‐673 to ‐668 bp) was PCR amplified using primers (GSP8, Table S1) containing *Sma*I and *Xho*I restriction sites (ADC‐P1) and cloned into the pAbAi vector to construct the bait. Both the effector vector and reporter vector were co‐transformed into yeast strain Y1H Gold following the manufacturer's instructions (Clontech). The transformed cells were spread on SD/‐Ura/‐Leu medium added with (0 or 300 ng/mL) AbA, and incubated for 3 days at 30 °C. Both positive (pGAD‐p53+ p53‐AbAi) and negative (pGADT7‐AD + P1) controls were included and processed in the same way.

### Transient expression analysis

For transient expression assays using *Arabidopsis* protoplasts, The *PbrADC* promoter region was amplified using primers (GSP9, Table S1) containing *Pst*I and *BamH*I restriction sites and inserted into pGreenII 0800‐LUC to generate the reporter plasmids. The MYB *cis*‐element in P1 was mutated by PCR (GSP10, Table S1). The coding region of *PbrMYB21* (GSP11, Table S1) was digested with *Sma*I and *Xho*I from the prey vector and inserted into pGreenII 62‐SK linearized with the same enzymes, generating an effector plasmid. The assays for transient expression in protoplasts were performed as described (Agarwal *et al*., 2006). All the plasmids used in this assay were extracted with QIA–GEN plasmid Midi Kit. The promoter activity was expressed as the ratio of LUC/REN. The protoplasts co‐transformed with normal reporters and pGreenII 62‐SK vector without PbrMYB21 was considered as control; LUC/REN ratio of which was set as 1.

For transcriptional activity analysis, a 35S‐UAS‐GUS reporter construct was obtained from Xia Li. For this purpose, the coding sequence of PbrMYB21 was amplified and ligated to the pYF503 vector, generating an effector GDBD‐PbrMYB21 construct. The effector construct, empty control (pYF503; designated as GDBD), and reporter plasmid 35S‐UAS‐GUS were transformed into *A. tumefaciens* strain GV3101 cells, and the two effectors were co‐infiltrated with the reporter 35S‐UAS‐GUS into *N. benthamiana* leaves as described previously (Voinnet *et al*., 2003). The abaxial surfaces of tobacco leaves were then kept in an incubator for 2 days, followed by histochemical GUS staining. For GUS staining, the tobacco leaves were submersed in GUS reaction mix [0.05 mm sodium phosphate buffer, pH 7.0, 1 mM X‐gluc and 0.1% (v/v) Triton X‐100], and were incubated at 37 °C overnight, followed by a 70% (v/v) ethanol wash.

### Southern and Western blot analysis

Genomic DNA was extracted from transgenic tobacco leaves using a DNeasy Plant Mini Kit (Qiagen GmbH, Hilden, Germany). For Southern blotting analysis, 15 μg of DNA was digested overnight with the restriction enzyme *Kpn*I without cleavage sites in *PbrMYB21*. The *PbrMYB21* overexpressing vector was used as a positive control. The digested products were fractionated on a 0.8% agarose gel, followed by transfer to Hybond‐N+ nylon membrane (Amersham Pharmacia Biotech, NJ, USA). Hybridization was carried out using a DIG‐High Prime DNA Labeling and Detection Starter Kit II according to the method of manufacturer's instruction (Roche, Germany).

A total of 1 g of transgenic tobacco plants for each sample were ground in the buffer containing 20 mM Tris (pH 7.4), 100 mM NaCl, 0.5% NonidetP‐40, 0.5 mM EDTA, 0.5 mM phenylmethylsulfonyl fluoride and 0.5% Protease Inhibitor Cocktail (Sigma‐Aldrich). PbrMYB21 protein levels were determined by protein gel blotting using an anti‐His antibody, and the protein extracts were separated on a 10% SDS‐PAGE gel and transferred to polyvinylidene difluoride membranes (Millipore) using an electrotransfer apparatus (Bio‐Rad). The membranes were incubated with anti‐His (Sigma‐Aldrich) primary antibodies and then peroxidase‐conjugated secondary antibodies (Abcam) before visualization of immunoreactive proteins using an ECL detection kit (Millipore). Actin served as a protein loading control.

### Emsa

EMSAs were performed using the LightShift Chemiluminescent EMSA Kit (Pierce) according to the manufacturer's protocol and as described by Hu *et al*. (2016). For the His‐PbrMYB21 construct, the CDS of PbrMYB21 was amplified and inserted into pCzn1‐His vector to generate the recombinant His‐PbrMYB21 protein. The recombinant protein was expressed and purified using Ni‐IDA resin according to the manufacturer's instructions. The binding activity of the protein was analysed using an oligonucleotide containing CAACTG motif present in the *PbrADC* promoter, labelled using the biotin 3′ End DNA Labeling Kit (Thermo Fisher Scientific). The same fragment without biotin labelling was used as a competitor. In addition, a mutated fragment of the probe was also labelled, in which CAACTG was replaced by CGCTGG. The binding reaction was incubated with the fusion protein in a 20 μL reaction solution at room temperature for 20 min. The DNA–protein complexes were separated on 6.5% nondenaturing polyacrylamide gels and electrophoretically transferred to a nylon membrane (GE Healthcare), and detected following the manufacturer's instructions. After UV cross‐linking, migration of the biotin‐labelled probes on the membrane was detected using streptavidin–horseradish peroxidase conjugates that bind to biotin and the chemiluminescent substrate according to the manufacturer's instructions. This experiment was performed three times.

### Statistical analysis

The data were statistically processed using the SAS software package (SAS Institute); analysis of variance (ANOVA) was used to compare the statistical difference based on *t*‐test at the significance levels of *P* < 0.05 (*), *P* < 0.01 (**), and *P* < 0.001 (***). Three technical replicates were used for each sample, and the data are shown as means ± standard errors (SE; *n* = 3). Three biological replicates were used for each of the genotypes, the wild type, OE‐9, OE‐17, OE‐20, pTRV‐PbrMYB21 plants and pTRV plants.

## Conflict of interest

The authors declare no conflict of interests.

## Supporting information


**Figure S1.** A phylogenetic tree was constructed using amino acid of PbrMYB21 and MYBs of other plants, such as *Malus domestica*,* Pyrus communis*,* Citrus sinensis* and *Glycine max*.
**Figure S2.** Regeneration of tobacco leaf discs and molecular characterization of the transgenic tobacco plants.
**Figure S3.** Southern blotting analysis of *PbrMYB21* in the three transgenic lines genome.
**Table S1.** Primer sequences used for cloning, subcellular localization, vector construction, transgenic confirmation and expression analysis
