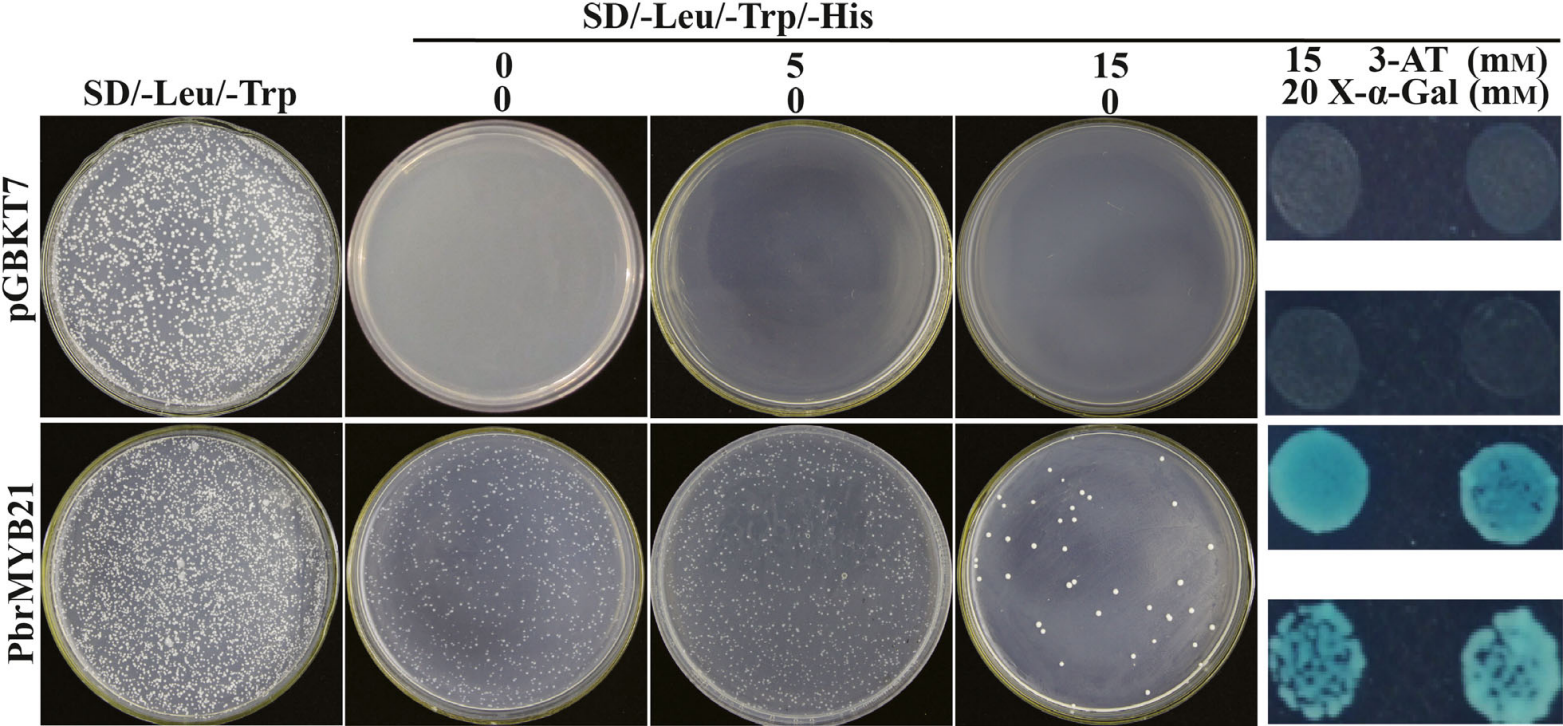# Corrigendum

**DOI:** 10.1111/pbi.13442

**Published:** 2020-08-09

The authors of Li *et al. *(2017) have identified error in Figure 4 after original publication.

See corrected figure below: