# Supplementary material for: PbrMYB21, a novel MYB protein of Pyrus betulaefolia, functions in drought tolerance and modulates polyamine levels by regulating arginine decarboxylase gene
Source: Plant Biotechnol J. 2017 Apr 1;15(9):1186–203. doi: 10.1111/pbi.12708 (PMC5552480; doi:10.1111/pbi.12708)
Supplement: Supplementary file 1 — Figure S1. A phylogenetic tree was constructed using amino acid of PbrMYB21 and MYBs of other plants, such as Malus domestica, Pyrus communis, Citrus sinensis and Glycine max. Figure S2. Regeneration of tobacco leaf discs and molecular characterization of the transgenic tobacco plants. Figure S3. Southern blotting analysis of PbrMYB21 in the three transgenic lines genome. Table S1. Primer sequences used for cloning, subcellular localization, vector construction, transgenic confirmation and expression analysis [file PBI-15-1186-s001.doc]

**Supporting information**

Figure S1. A phylogenetic tree was constructed using amino acid of PbrMYB21 and MYBs of other plants, such as *Malus domestica*, *Pyrus communis*, *Citrus sinensis* and *Glycine max*.

Figure S2. Regeneration of tobacco leaf discs and molecular characterization of the transgenic tobacco plants. (A-D) Regeneration processes of the tobacco leaf discs via *Agrobacterium*-mediated transformation of the overexpression vector. (E) Genomic PCR identification of the regenerated plants using specific primers of *PbrMYB21*. M, DNA marker (DL 2000). +, positive control (gene plasmid). -, negative control (untransformed plants). Numbers on the top of the two gel panels indicate the transgenic lines. (F) Semi-quantitative RT-PCR analysis of the mRNA levels of *PbrMYB21* in the twelve transgenic lines and wild type (WT). (G) Western blotting analysis of *PbrMYB21* proteins in the wild-type and transgenic tobacco. (H) Semi-quantitative RT-PCR analysis of the transcript levels of *PbrMYB21* in *Pyrus betulaefolia* silenced plants and wild type (WT). *Tublin* and *Ubiquitin* were analysed in parallel as internal reference controls for *Pyrus betulaefolia* and tobacco, respectively, to normalize expression levels. *ACTIN* served as a protein loading control for transgenic tobacco.


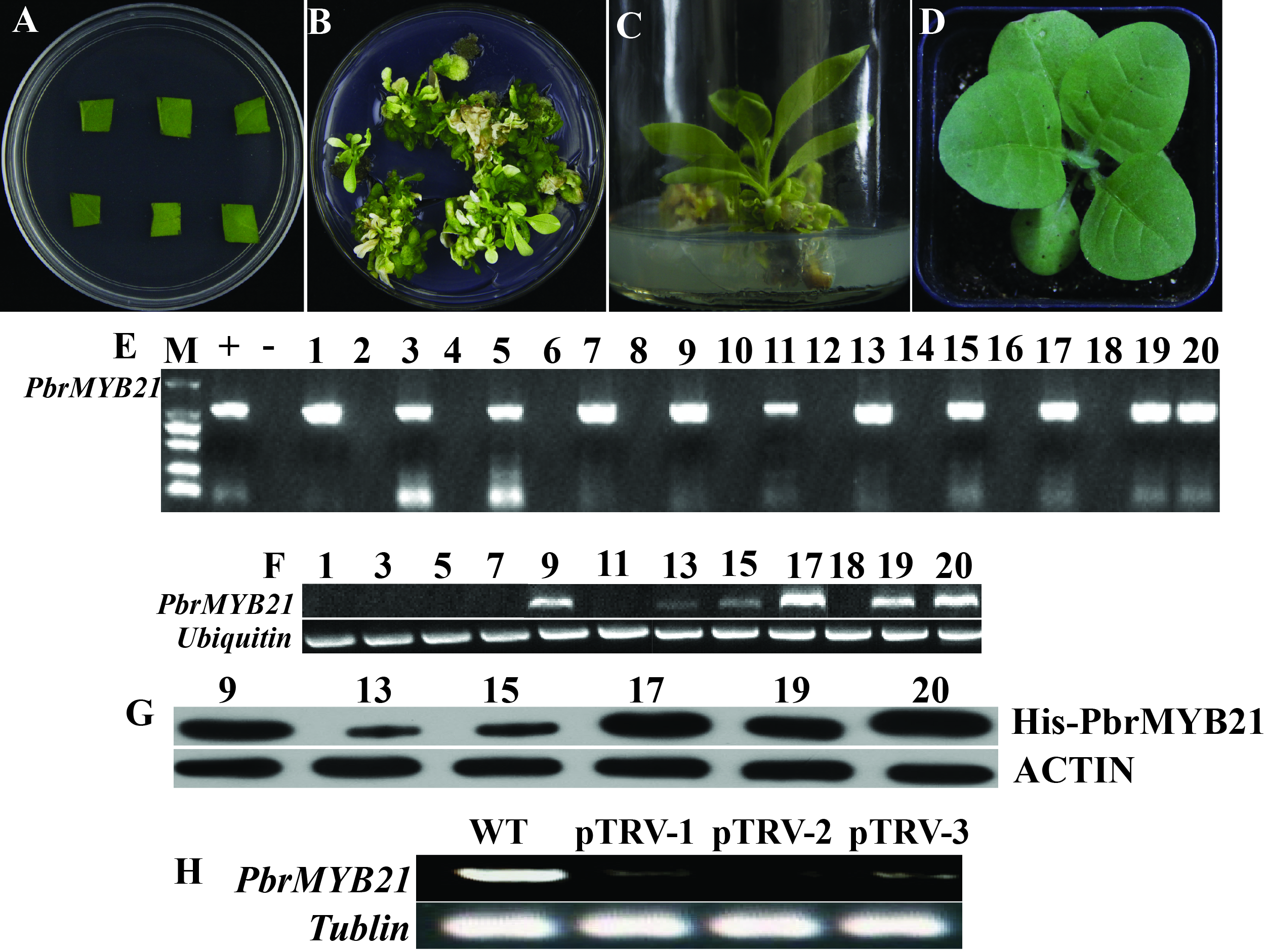


Figure S3. Southern blotting analysis of *PbrMYB21* in the three transgenic lines genome. M, DNA marker (AL2000). +, positive control (gene plasmid). -, negative control (untransformed plants). 9, OE-9; 17, OE-17, 20, OE-20. In total 15 µg of genomic DNA digested with KpnI was fractionated on a 0.8% agarose gel, blotted to a nylon membrane, and hybridized as described in the materials and methods. The molecular size of the hybridized signals is indicated on the left (kb).


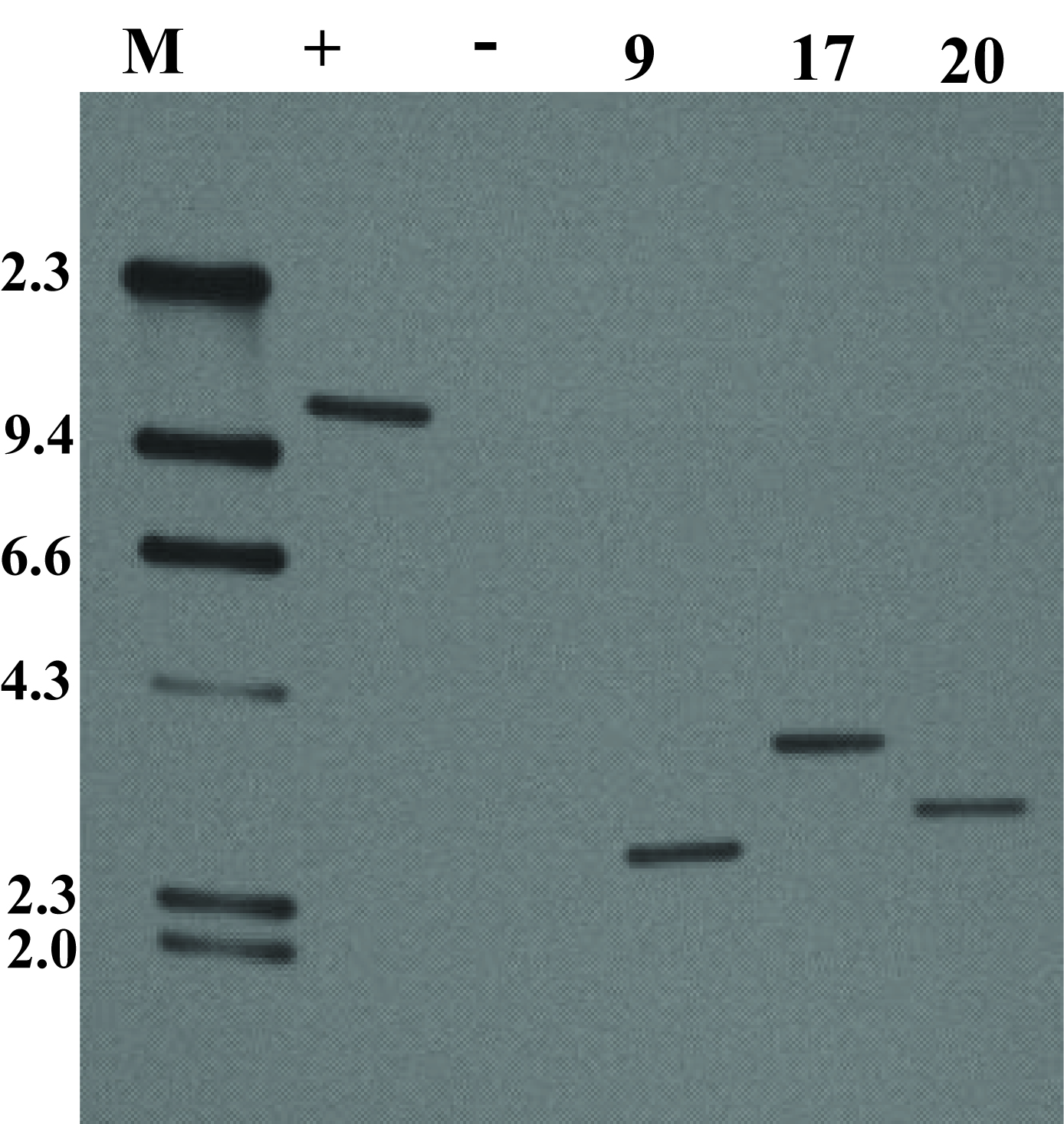


Table S1 Primer sequences used for cloning, subcellular localization, vector construction, transgenic confirmation and expression analysis.

| **Genes** | **Primers** | **Sequences (**5ʹ-3ʹ**)** | |
| --- | --- | --- | --- |
|  |  | **Forward** | **Reverse** |
| *PbrMYB21* | GSP1 | GGTCTAAAATTGCGCAGCAT | TCGACTCGATGTTCAGTTGG |
| *PbrMYB21* | GSP2 | ATGGCTGCTCCTAGAAACCCTAATGAA | TCACATCCCGTCCATGTTCCATAAATTGTC |
| Tublin  *PbrMYB21*  *PbrMYB21*  *PbrMYB21*  *PbrMYB21* | GSP3  GSP4  GSP5  GSP6 | TGGGCTTTGCTCCTCTTAC  GGATCCATGGCTGCTCCTAGAAACCCTAATG(*BamH* I site is underlined)  GAAGATCTATGGCTGCTCCTAGAAACCCTAATG (*Bgl* II site is underlined)  GAAGATCTATGGCTGCTCCTAGAAACCCTAATG (*Bgl* II site is underlined)  GAATTGGCGAACCACGGCGAAGGCCATTGG (*Ecor*I site is underlined) | CCTTCGTGCTCATCTTACC  CCATGGCATCCCGTCCATGTTCCATAAATTGTC (*Nco*I site is underlined)  GACTAGTCATCCCGTCCATGTTCCATAAATTGTC (*Spe*I site is underlined)  GGTGACCGTGATGGTGATGGTGATGCATCCCGTCCATGTTCCATAAATTGTC (*BstE*II site)  CTCGAGGCACGCTGTTCGACTCGATGTTCAG (*Xho*I site is underlined) |
| *PbrMYB21*  *PbrADC*  *PbrADC*  *PbrADC*  *PbrMYB21*  *NtERD10C*  *NtLEA5*  *NtP5CS*  *NtADC1*  *NtADC2*  *NtSAMDC*  *PbrP5CS*  PbrSAMDC  PbrLEA5  PbrERD10C  PbrADC | GSP7  GSP8  GSP9  GSP10  GSP11 | GGATCCATGGCTGCTCCTAGAAACCCTAATG (*BamH*I site is underlined)  CCCGGGCGCACTCCTCTCCCTCTCGCGGCG (*Sma*I site is underlined)  CTGCAGCTCACGAGAAAGCCAGTTGAACCACG (*Pst*I site is underlined)  CTGCAGCTCACGAGAAAGCCGCTTGAACCACG (*Pst*I site is underlined)  CCCGGGATGGCTGCTCCTAGAAACCCTAATG (*Sma*I site is underlined)  ACGTGGAGGCTACAGATCGTGGTTTG  CTCTAACTCCAAACTCATCTCTGC  TTCCAGACGTCTTCAGGCAC  CTTGCTGATTACCGCAATTTATC  GCCGGCCCTAGGTTGTTGTGTAGATG  ATTGGTTTTGAAGGTTTTGAGAAG  AGACGTTAAGCGCCTCGTTA  AATCCGGCATCAGAAACATC  TCGTCGACGGTTTCTCTAGC  GCCCACTGATTACCCAACTG  ACATGCCGTATCTGGTGACA | CCATGGCATCCCGTCCATGTTCCATAAATTGTC (*Nco*I site is underlined)  CTCGAGAGGGTTGGGCCGGTGGGACCCGCC (*Xho*I site is underlined)  GGATCCGTCACCTGAGACAAATGGAAACT  GGG (*BamH* I site is underlined)  GGATCCGTCACCTGAGACAAATGGAAACTGGG (*BamH* I site is underlined)  CTCGAGCATCCCGTCCATGTTCCATAAATTGTC (*Xho*I site is underlined)  TCTCCACTGGTACAGCCGTGTCCTCAC  CAAAACCCCAGATTCAAGAC   TCATATCCGGCCTGTTGAGC  CCTTACTGCAGGCTTTTCATCTA  AGCGAACAACAAGAGGCAGCTGAAGCC  TCACGTCTTGTACTTTGAGAGACAG  TTCAGCTGCTCACAAAGTGC  AACTGCATTCCCTTCGATTG  TCTGGGATTCGTAGCACCTC  GCCCACTGATTACCCAACTG  TCCGAAACAACATCGTCGTA |
| ***Ubiquitin*** |  | AGCTACATGACGCCATTTCC | CCCTGTAAAGCAGCACCTTC |
